# Temporally and spatially resolved molecular profiling in fingerprint analysis using indium vanadate nanosheets-assisted laser desorption ionization mass spectrometry

**DOI:** 10.1186/s12951-023-02239-w

**Published:** 2023-12-10

**Authors:** Yanli Zhu, Jikai Wang, Chengxiao Fu, Shuangquan Liu, Pragati Awasthi, Pengfei Zeng, Danjun Chen, Yiyang Sun, Ziyi Mo, Hailing Liu

**Affiliations:** 1 Hunan Province Cooperative Innovation Center for Molecular Target New Drug Study, Institute of Pharmacy & Pharmacology, Hengyang Medical School, University of South China, Hengyang, Hunan 421001 P. R. China; 2 School of Resources and Environment, Hunan University of Technology and Business, Changsha, Hunan 410205 P. R. China; 3 The First Affiliated Hospital, Department of Clinical Laboratory, Department of Pharmacy, Hengyang Clinical Pharmacology Research Center, Hengyang Medical School, University of South China, Hengyang, Hunan 421001 P. R. China; 4 State Key Laboratory of Silicon Materials & School of Materials Science and Engineering, Zhejiang University, Hangzhou, Zhejiang 310058 P. R. China; 5 Department of Respiratory and Critical Care Medicine, Renmin Hospital of Wuhan University, Wuhan, Hubei 430060 P. R. China

**Keywords:** Indium vanadate nanosheets, Mass spectrometry imaging, Ionization mechanism, Chemical mapping, Fingerprint analysis

## Abstract

This study presents the first-ever synthesis of samarium-doped indium vanadate nanosheets (IVONSs:Sm) via microemulsion-mediated solvothermal method. The nanosheets were subsequently utilized as a nano-matrix in laser desorption/ionization mass spectrometry (LDI-MS). It was discovered that the as-synthesized IVONSs:Sm possessed the following advantages: improved mass spectrometry signal, minimal matrix-related background, and exceptional stability in negative-ion mode. These qualities overcame the limitations of conventional matrices and enabled the sensitive detection of small biomolecules such as fatty acids. The negative-ion LDI mechanism of IVONSs:Sm was examined through the implementation of density functional theory simulation. Using IVONSs:Sm-assisted LDI-MS, fingerprint recognitions based on morphology and chemical profiles of endogenous/exogenous compounds were also achieved. Notably, crucial characteristics such as the age of an individual’s fingerprints and their physical state could be assessed through the longitudinal monitoring of particular biomolecules (e.g., ascorbic acid, fatty acid) or the specific biomarker bilirubin glucuronide. Critical information pertinent to the identification of an individual would thus be facilitated by the analysis of the compounds underlying the fingerprint patterns.

**Graphical Abstract:**

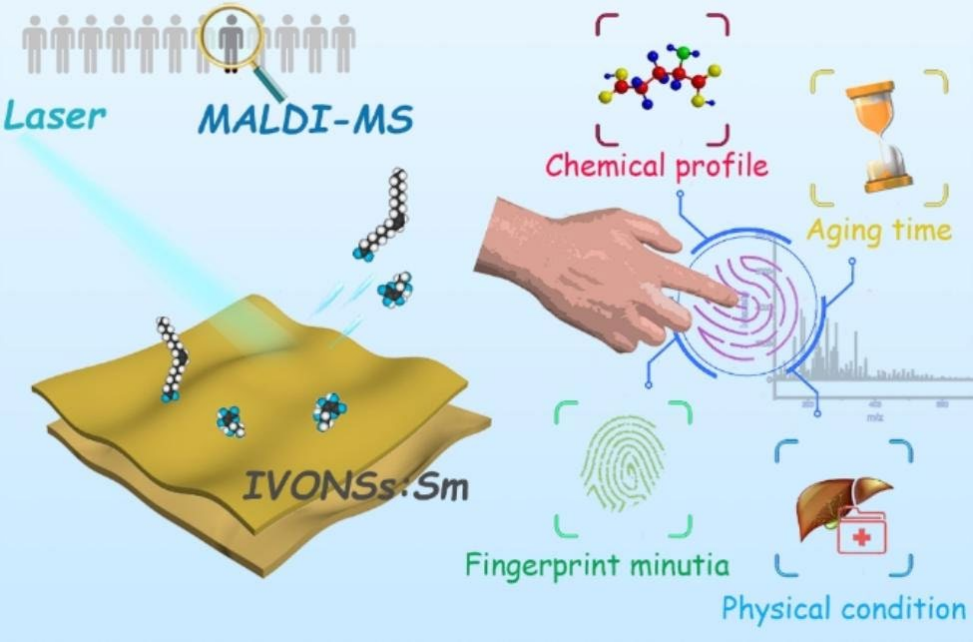

**Supplementary Information:**

The online version contains supplementary material available at 10.1186/s12951-023-02239-w.

## Introduction

Matrix-assisted laser desorption/ionization mass spectrometry (MALDI-MS) has garnered considerable scientific interest due to its advantageous features such as high throughput, rapid analysis, and robustness against high salt concentrations. However, the effective utilization of MALDI-MS for the analysis of low-molecular-weight (LMW) compounds has been hindered by certain limitations, including suppressed signal intensity of the analyte and illegibility of the MS spectrum [1, 2]. Because the most widely used organic matrices like α-cyano-4-hydroxycinnamic acid (CHCA) and 9-aminoacridine (9-AA), generate matrix-related background in the MS region below m/z 700, interfering with the MS signal of LMW analytes [3, 4]. Besides, the “sweet spot” phenomenon resulting from the heterogeneous co-crystallization leads to inferior reproducibility [5, 6]. Unlike traditional MALDI-MS, surface-assisted laser desorption/ionization mass spectrometry (SALDI-MS) does not require any organic matrix, benefiting LMW compound detection with negligible matrix-related interference [7]. In the last decades, multifarious nanomaterials (e.g. porous silicon [8, 9], carbon-based materials [10–12], metal oxides [13–16], metal-organic frameworks [17, 18], and metal nanomaterials [19, 20]) several techniques have been developed for the fabrication of SALDI nano-matrices. The efficacy of these nano-matrices has been extensively demonstrated in several applications of SALDI-MS. For instance, Pleskunov et al. reported niobium nanoparticles based SALDI-MS method for multiple phospholipids analysis on mouse brain tissue [21]. Minhas et al. prepared nanostructured silicon SALDI-MS to detect anabolic doping agents and their metabolites in saliva and urine [22]. Chu et al. fabricated a metal nanoparticle-based sandwich immunosorbent sensing platform for the SALDI-MS detection of viruses and viral nonstructural proteins [23]. For the detection of p-phenylenediamine, Peng et al. introduced a novel magnetic single-layer nano-MXene that could function concurrently as an enrichment material and SALDI matrix [24]. Despite the advancements made in SALDI nano-matrices, certain limitations persist in disruptive carbon or metal clusters, unintended surface fouling, inadequate physicochemical stability, and indistinct alkali adduct ions. Therefore, investigating a novel nano-matrix with features of low background, good stability, and high ionization efficiency is still of great value. Recently, the evidentiary significance of fingerprint recognition utilizing minutiae and the chemical composition of endogenous/exogenous chemicals has been substantiated [25, 26]. Such chemical information provides personal details like dietary habit, physical condition, and living style [27, 28]. Furthermore, determining the age of the fingerprint holds forensic importance. The identification of the age of fingerprints can achieve the determination of the temporal scope of criminal cases [29, 30]. However, there was a rare approach to determine the chemicals and fingerprint age simultaneously.

In this research, we synthesized samarium-doped indium vanadate nanosheets (IVONSs:Sm) as a novel nano-matrix for the negative-ion LDI-MS analysis of LMW molecules. The as-synthesized IVONSs:Sm exhibited properties like enhanced optical absorption, high charge mobility, and a large surface area, which not only contributed to an efficient absorption and transfer of laser energy but also facilitated the deprotonation of the analytes. Generally speaking, better performance in analyzing of LMW compounds could be obtained in negative-ion mode without multiple adduct peaks [31], hence IVONSs:Sm-assisted LDI-MS in negative-ion mode was endowed with enhanced MS signal and legible MS spectrum. Consequently, IVONSs:Sm-assisted LDI-MS with good sensitivity and repeatability was successfully applied in fingerprint analysis. Along with the ridge pattern, chemicals including endogenous (e.g., fatty acids) and exogenous (e.g., drug residues) molecules on fingerprints could be identified via the IVONSs:Sm-assisted LDI-MS imaging technology. Moreover, additional trials investigating the determination of fingerprint age and the assessment of biomarkers related to hepatic injury have demonstrated the potential efficacy of this method as a viable option in both forensic and clinical contexts.

## Materials & methods

### Chemicals

All starting materials, unless mentioned otherwise, were obtained from commercial suppliers and used directly. Ammonium metavanadate (NH_4_VO_3_, 99.95%), hexadecylpyridinium bromide (CDPB, 96%), lauric acid (LA, analytical standard), myristic acid (MA, analytical standard), palmitoleic acid (PA, analytical standard), and oleic acid (OA, analytical standard), n-butanol (C_4_H_10_O, 99%), n-octane (C_4_H_10_O, 96%), isopropanol (C_3_H_7_OH, 99.7%), nitric acid (HNO_3_, 70%), and acetonitrile (CH_3_CN, HPLC grade) were purchased from Aladdin Reagent Co., Ltd. (Shanghai, China). Indium nitrate (In(NO_3_)_3_, 99.99%), samarium nitrate (Sm(NO_3_)_3_, 99.99%), potassium thiocyanate (KSCN, 99.95%) were purchased from Macklin Reagent Co., Ltd. (Shanghai, China). Methanol (CH_3_OH, anhydrous), ethanol (C_2_H_5_OH, anhydrous) were purchased from Sinopharm Chemical Reagent Co., Ltd. (Shanghai, China). α-Cyano-4-hydroxycinnamic acid (CHCA, 99%) and 9-aminoacridine (9-AA, 97%) were obtained from Sigma-Aldrich Reagent, Co., Ltd. (St. Louis, USA). Bromoethane (C_2_H_5_Br, 99%) was provided by Fuyu Chemical Reagent Factory (Tianjin, China). Chemically converted graphene (thickness 0.8–1.2 nm) and nano-sized CeO_2_ (diameter 4–8 nm) were purchased from Ruixi Biological Technology Co., Ltd. (Xi’an, China). Hand lotion (Blue Moon, Co., Ltd. Guangdong, China) and acne cream (Liangfu Pharmaceutical Co., Ltd. Shandong, China) used in exogenous LMW compounds determination were purchased from a local supermarket and pharmacy. Ultrapure water (≥ 18.2 MΩ•cm) produced by an Aqua-pro water system (Aquapro, Chongqing, China) was used throughout the experiment.

### Apparatus

TEM images of the nano-matrices, including IVONSs:Sm, graphene and CeO_2_, were collected by transmission electron microscope (FEI Tecnai F20) at 200 kV. XRD patterns were recorded by powder X-ray diffractometer (D8 Advance Bruker) from 10° to 80° (4°/min) using Cu-Ka radiation (λ = 0.1542 nm). Diffuse reflectance UV-visible spectra were acquired on UV-vis spectrometer (PerkinElmer UV3600). XPS spectra were measured using X-ray photoelectron spectrometer (Thermo ESCALAB 250XI), which was calibrated based on the binding energy of the C 1s peak at 284.8 eV. Vanadium element analysis of IVONSs:Sm deposited on the copper conductive tape were qualitatively measured by ICP-MS (Agilent 8900) with microwave digestion system. SEM observation of fingerprint samples were performed on scanning electron microscopy (ZEISS Sigma 300) using 15 kV beam energy. AFM and SPV images of indium vanadate nanosheets were taken on atomic force microscope (Bruker Dimension ICON). Images were acquired at scan rates of 1.0 Hz, and the tip lift height was 120 nm at tapping mode for potential mapping. The SPV images were derived from the changes of contact potential difference before and after UV light irradiation. The photothermal effect of the nano-matrices on glass slides was performed under 405 nm laser irradiation (2 W/cm^2^), the infrared thermographic images and temperature were determined with an infrared thermal imaging camera. The hydrophilicity/hydrophobicity of the nano-matrices was analyzed by measuring the aqueous contact angle using optical contact angle measuring and contour analysis systems (Dataphysics OCA20). Determination of fatty acids in fingerprint samples was carried out in HPLC-ESI-MS (Agilent 1290 Infinity/6460 Triple Quadrupole). All the MALDI-MS and tandem MS experiments were carried out on a mass spectrometer (Bruker Ultraflextreme MALDI TOF/TOF) with neodymium-doped yttrium aluminium garnet solid-state laser (Nd:YAG laser, 355 nm wavelength) in positive- or negative-ion mode.

### Synthesis of IVONSs:Sm

The microemulsion-assisted solvothermal method was employed to synthesize IVONSs:Sm and pure IVONSs. In a typical procedure for IVONSs:Sm synthesis, two types of microemulsion (ME_In_ and ME_V_) with different aqueous phases were prepared. Both ME_In_ and ME_V_ contained 3.5 mmol of emulsifier, CPDB, 25 mL of co-emulsifier, n-butanol, and 100 mL of the oil phase, n-octane. For the ME_In_ system, the microemulsion was vigorously magnetically stirred for 150 min after being mixed with 5 mL of an aqueous solution containing indium nitrate (50 mM) and samarium nitrate (2.5 mM). Meanwhile, the aqueous solution of ammonium metavanadate (5.5 mL, 50 mM) was added to the ME_V_ system under continuous stirring until the microemulsion was homogeneous. Afterwards, the ME_In_ system was added dropwise into the ME_V_ system with stirring magnetically for 2 h at room temperature, achieved by the addition of nitric acid (2 M) to adjust the pH value to 1–2. The mixed microemulsion was transferred into a Teflon-lined stainless-steel autoclave for solvothermal treatment at 170 °C for 20 h, followed by cooling to room temperature naturally. Finally, IVONSs:Sm were obtained after centrifugation and washed with deionized water and ethanol. For pure IVONSs synthesis, the synthetic procedure was similar except for the removal of the samarium nitrate reagent.

### Mass spectroscopy with nano-matrices and organic matrices

In the preliminary application test, we used a mixture of fatty acids as model LMW molecules to evaluate the SALDI-MS performance of the nano-matrices (i.e. IVONSs:Sm, graphene, CeO_2_) and traditional organic matrices (i.e. CHCA, 9-AA). Here, four fatty acids were dissolved in methanol (1000 µM) and subsequently diluted to a specified concentration as required for specific experiments. The organic matrix CHCA (10 mg/mL) was dissolved in CH_3_CN/water (2:1, v/v) containing 0.1% trifluoroacetic acid. 9-AA was prepared at 10 mg/mL in CH_3_CN/water (1:1, v/v). The IVONSs:Sm and pure IVONSs suspensions were prepared in ethanol/H_2_O (3:1, v/v) and then sonicated for 10 min, forming a nano-matrix suspension with a concentration of 1.0 mg/mL. The suspension of nano-matrix graphene or CeO_2_ was prepared according to previous literature. Specifically, graphene was dispersed in ethanol at 1.0 mg/mL with the aid of ultrasonication, and CeO_2_ was dispersed in isopropanol and formed a homogeneous suspension at a concentration of 25 mg/mL. A total of 1 µL of matrix suspension was dropped onto the ground-steel sample target with 384 spots and then air-dried, followed by 1 µL of analyte solution at the specified concentration being deposited on top of the matrix. Additionally, the backgrounds of matrices of CHCA, 9-AA, and IVONSs:Sm were also collected under identical mass spectrometry conditions. For the IVONSs:Sm-assisted LDI-MS spectra of LA and OA mixtures in the presence of KSCN, different concentrations of KSCN were blended with the isovolumetric IVONSs:Sm suspensions beforehand. All the mass spectrometric data were acquired using the Bruker Ultraflextreme MALDI TOF/TOF MS equipped with a Smart-Beam II Nd:YAG laser (355 nm wavelength, 50 μm laser spot, 2000 Hz), either in the reflective positive- or negative-ion mode. Each MS spectrum was acquired as an accumulation of 1000 laser shots. Mass calibrations were performed externally using the MS peaks of CHCA for LMW molecule analysis. The laser irradiation power was controlled with an attenuation filter. The laser fluence was shown as a percentage of the laser output and set at 75% laser output for LMW compounds analysis, except where noted. The MALDI-MS instrument was controlled via the flexControl software (Bruker Daltonics, Inc., Germany). MS spectra and images were processed via the Bruker Daltonics flexAnalysis software.

### Fingerprint samples preparation and analysis

All the fingerprint samples were volunteered by laboratory members. The fingerprints were laid onto the surfaces of the windshield (glass), ID card (plastic), and knife (metal) for 5 s after rubbing the fingers on their forehead once. Fresh fingerprints were immediately sprayed with IVONSs:Sm in bromoethane (5 mg/mL) using a handheld electronic sprayer (NVisions Co., Ltd., China) with a 10 cm spacing distance. The flow rate of spraying was 0.01 mL/s, and the spraying time of 30 s was used for the nano-matrix deposition. A nitrogen-blow procedure was used after 180 s of drying at room temperature to detach the loosely sorbed IVONS:Sm. Subsequently, a double-sided copper foil tape was uniformly affixed to the nano-matrix-deposited fingerprint sample for 15 min. The double-sided copper foil tape was stripped from the surface of the fingerprint samples and tailored to an appropriate size, then adhesively stuck to an indium tin oxide-coated glass slide for subsequent SALDI-MS analysis. In addition, the surface microtopography and elemental analysis of extracted fingerprints were carried out using scanning electron microscopy (ZEISS Sigma 300) equipped with X-ray spectrometer attachments after the metal sputtering-treated procedure. For fingerprint aging time determination, fingerprint samples were stored in the constant temperature and humidity test chamber (YP-150GSP, Taisite Co., Ltd., China) for various time points to simulate typical ambient conditions. The temperature, relative humidity, and lighting conditions were controlled and monitored. The fingerprint age of the simulated fingerprint samples was predicted based on the equation as follows: T = (t_OA_ + t_AA_ + t_TA_)/3, where T stood for the fingerprint age, and t_OA_, t_AA_, and t_TA_ referred to the aging time of the three specific analytes, oleic acid (OA), ascorbic acid (AA), and threonic acid (TA), respectively. The aging time of each specific analyte was respectively and semi-quantitatively evaluated from the experimental fit curves between time-dependent MS intensity ratios (R_OA_, R_AA_, R_TA_) and storage periods. To detect exogenous LMW compounds on fingerprints, fingerprint samples were collected after washing hands with lotion or rubbing the fingertips on volunteers’ faces coated with acne cream. IVONSs:Sm-assisted LDI-MS imaging data were acquired using the Bruker Ultraflextreme MALDI TOF/TOF MS equipped with a Smart-Beam II Nd:YAG laser (355 nm wavelength, 50 μm laser spot, 2000 Hz) in negative-ion mode. The data acquisition was performed at the mass range of m/z 100–800 using a spatial resolution of 80 μm and collecting a total of 500 laser shots per pixel. All the chemical maps of LMW molecules were reconstructed and visualized using the Bruker Daltonics flexImaging software. The confirmation of LMW compounds was achieved by matching the molecular weight in the online database (https://webbook.nist.gov/chemistry/) and previously published results [32–36].

## Results and discussion

### Characterization and LDI-MS performance of IVONS:Sm

**Fig. 1:**
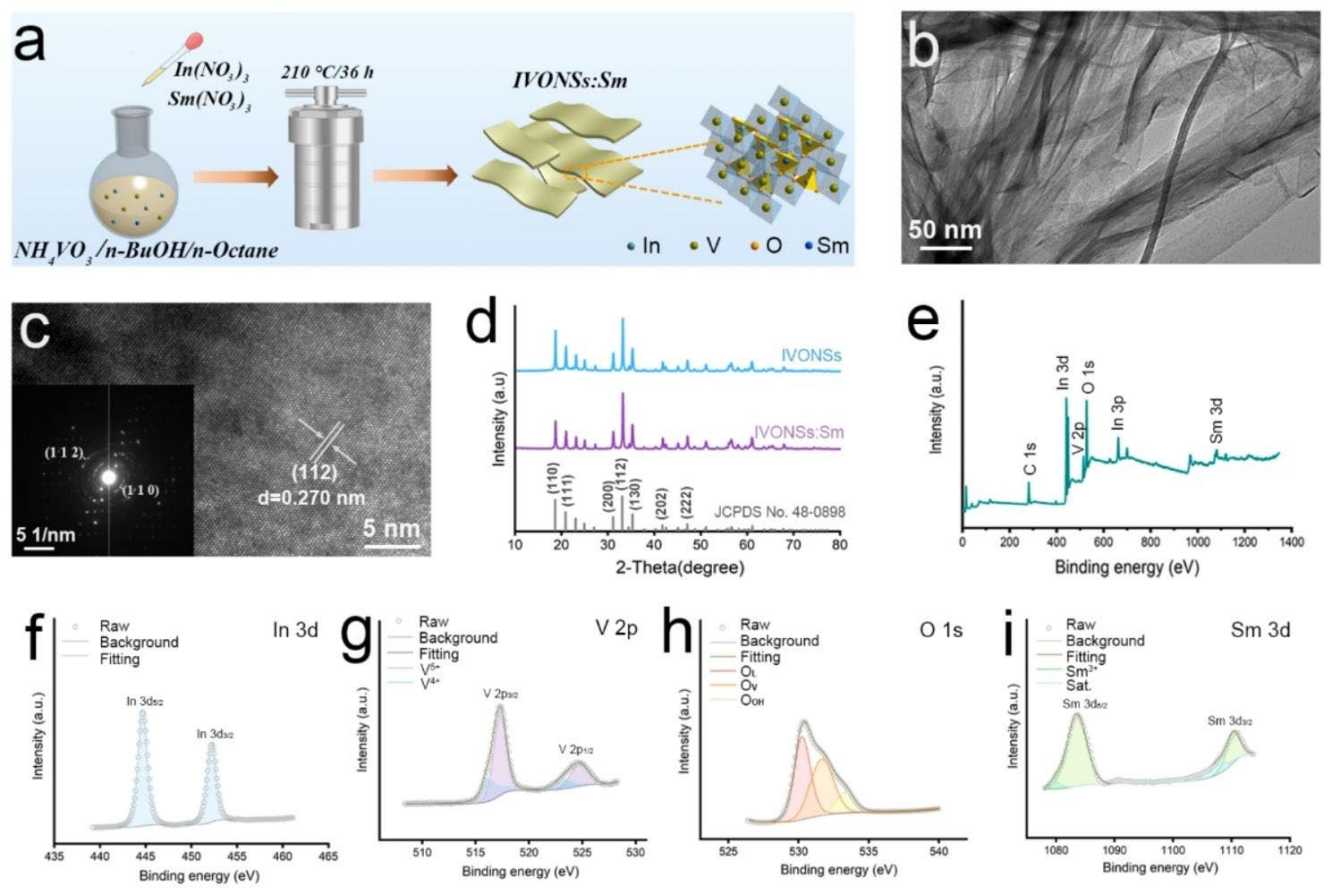
(**a**) Schematic illustration of IVONSs:Sm preparation based on the microemulsion-mediated solvothermal method. (**b**) TEM image, (**c**) HRTEM image, and SAED pattern (inset) of IVONSs:Sm. (**d**) XRD spectra of IVONSs:Sm and pure IVONSs. XPS analysis of IVONSs:Sm: (**e**) Survey spectrum, (**f**) In 3d, (**g**) V 2p, (**h**) O 1s, and (**i**) Sm 3d

As depicted in Fig. 1a, the preparation of IVONSs:Sm was carried out using the microemulsion-mediated solvothermal method. Transmission electron microscopy (TEM) analysis revealed that the as-synthesized IVONSs:Sm were of translucence and corrugation, indicative of a sheet-like nanostructure which was conducive to the adsorption of LMW molecules (Fig. 1b). A lattice spacing of 0.270 nm in high resolution TEM (HRTEM) image was indexed to (112) plane of orthorhombic InVO_4_ (Fig. 1c). Additionally, the selected area electron diffraction (SAED) pattern confirmed the high degree of crystallinity of IVONSs:Sm (inset, Fig. 1c). Elemental analysis using energy dispersive spectrum (EDS) and EDS mapping further verified the presence and uniform distribution of In, V, O, and Sm elements in IVONSs:Sm, with semiquantitative results closely aligning with the theoretical compositions (Figure S1a, b). Figure 1d exhibited the powder X-ray diffraction (XRD) patterns of IVONSs:Sm, the sharp diffraction peaks also revealed the good crystallinity of IVONSs:Sm and matched well with the standard orthorhombic-phase InVO_4_ (JCPDS No. 48–0898). It is noteworthy that Sm doping had no effect on the characteristic diffraction pattern of InVO_4_, and there was no impurity peak concerned with In_2_O_3_, V_2_O_5_ or other species. Beyond that, survey X-ray photoelectron spectroscopy (XPS) analysis confirmed the elements of In, V, O, and Sm without impurities (Fig. 1e). Additionally, high-resolution XPS spectra of In 3d, V 2p, O 1s, and Sm 3d provided detailed insights into the chemical composition and bonding states of the IVONSs:Sm (Fig. 1f ~ i). The high-resolved In 3d spectrum was characterized by an In 3d_5/2_ peak at 444.3 eV and an In 3d_3/2_ peak at 452.1 eV (Fig. 1f). For the V 2p spectrum, the fitted peaks at 516.9 eV and 525.0 eV were associated with V 2p_3/2_ and V 2p_1/2_ of V^5+^, respectively. While peaks located at 515.2 eV and 523.3 eV were assigned to V 2p orbitals of V^4+^, indicating V^5+^ might obtain electrons from nearby oxygen vacancies (Fig. 1g). As expected, O 1s spectrum revealed three deconvoluted peaks at 530.3, 532.1 and 533.6 eV, representing lattice oxygen (O_L_), vacancy oxygen (O_V_) and chemisorbed oxygen (O_OH_), respectively (Fig. 1h). In the components of Sm 3d peak, two peaks fitted at 1083.5 eV and 1110.9 eV were corresponded to Sm 3d_5/2_ and Sm 3d_3/2_ orbitals, respectively (Fig. 1i). Collectively, these characterization results provided evidence of the successful synthesis of IVONSs:Sm.

**Fig. 2:**
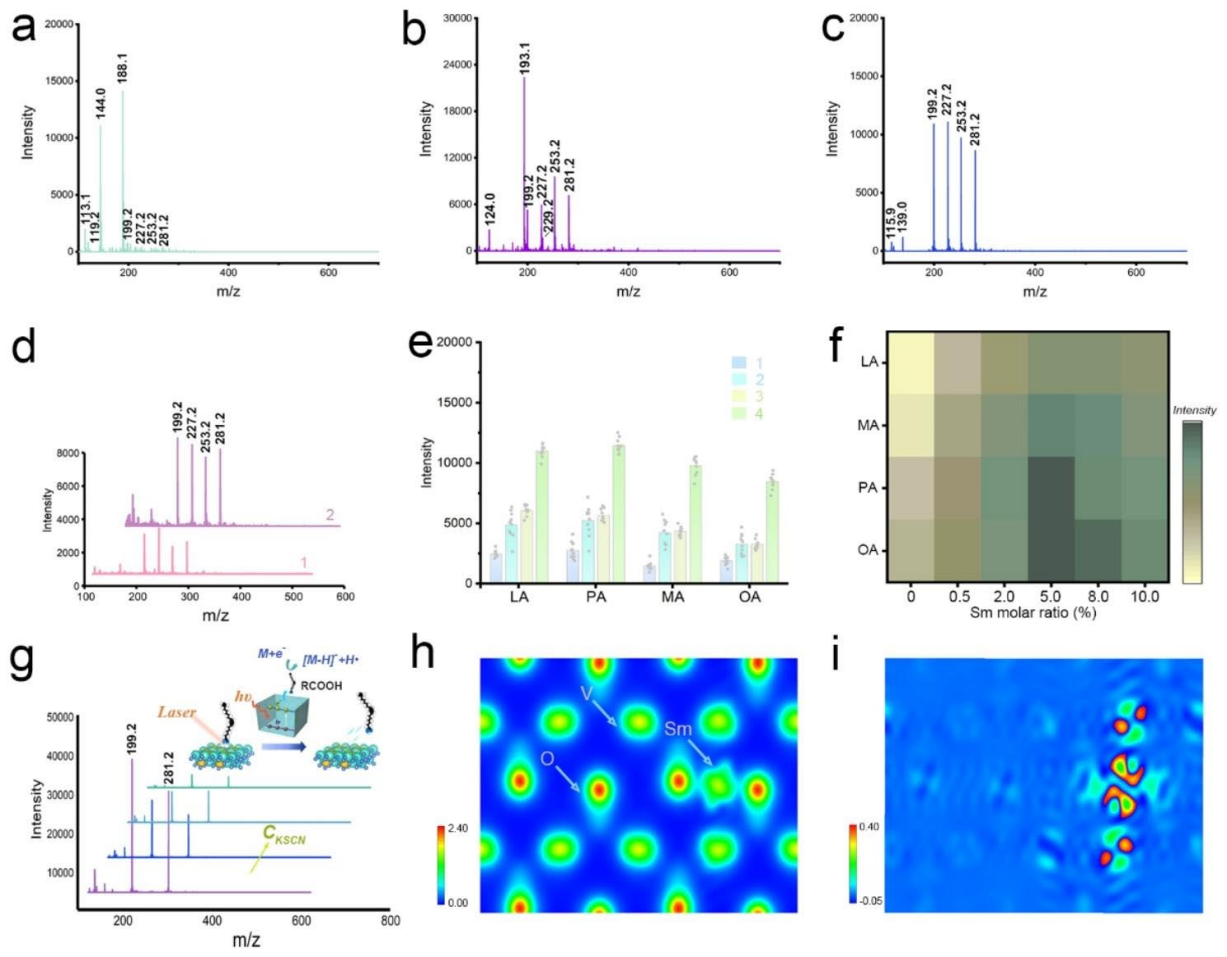
MS spectra of four fatty acids analyzed by using (**a**) CHCA, (**b**) 9-AA, and (**c**) IVONSs:Sm in negative-ion mode, respectively. (**d**) MS spectra of four fatty acids analyzed by using graphene (1) and CeO_2_ (2) in negative-ion mode. (**e**) MS intensity comparison among four fatty acids using different nano-matrices: graphene (1), CeO_2_ (2), IVONSs (3), and IVONSs:Sm (4). (**f**) Optimization of MS signal intensity versus the molar ratio of Sm doping amount for IVONS:Sm. (**g**) IVONSs:Sm-assisted LDI-MS spectra of LA and OA mixtures with different concentrations of KSCN in negative-ion mode, and the corresponding schematic diagram of the negative-ion LDI mechanism based on IVONSs:Sm (inset). (**h**) Electron density profile and (**i**) differential electron density profile of IVONSs:Sm

In order to evaluate the performance of IVONSs:Sm as a nano-matrix for LDI-MS analysis, the four most common fatty acids in organisms - lauric acid (LA), myristic acid (MA), palmitoleic acid (PA), and oleic acid (OA) - were selected as model analytes and analyzed with IVONSs:Sm, as well as traditional organic matrices (CHCA and 9-AA) in both positive- and negative-ion modes. When CHCA was utilized in the positive-ion mode, the cationic adducts and fragments of CHCA were found to be predominant, inhibiting the MS signals of the four analyte ions, including [M + H]^+^ and [M + Na]^+^, under these background signals (Figure S2a, Table S2). Additionally, Figure S2b also revealed a suppressive MS signal of the four fatty acids, indicating the inapplicability of 9-AA in positive-ion MALDI-MS analysis. As shown in Figure S2c, multiple positive-ion signals of fatty acids could be detected with less interference peak when IVONSs:Sm was used as a nano-matrix. However, it is noteworthy that accompanying the quasi-molecular ion peaks of fatty acids were the alkali and double alkali adduct ions of the analytes (Table S2), rendering the mass spectrum in positive-ion mode particularly complicated to interpret. These results imply that it may not be a good option to analyze fatty acids in the positive-ion mode. On the other hand, Fig. 2a and b demonstrated the MS spectra of the four fatty acids in negative-ion mode using CHCA and 9-AA as matrices. For CHCA, the matrix-related ions ([M − CO_2_ − H]^−^ at m/z 144.0 and [M − H] ^−^ at m/z 188.1) dominated, but suppressive analyte signals were observed. In addition, improved MS signal intensity and lower missing percentage of fatty acids were observed for the 9-AA matrix (Table S2). Nevertheless, a higher intensity of intrinsic matrix-related ion ([M-H]^−^ at m/z 193.1) still suppressed the MS signal of fatty acids. In contrast, an interference-free MS spectrum with deprotonated [M − H]^−^ ions of LA, MA, PA, and OA at m/z 199.2, 227.2, 253.2, and 281.2 was obtained in the range of m/z 150–600 with IVONSs:Sm as the nano-matrix (Fig. 2c), implying that IVONSs:Sm were more applicable in facilitating negative ionization for fatty acids. As a control, the performance of the three matrices without analytes was also evaluated (Figure S3). The results indicated that backgrounds with several intrinsic matrix-related peaks were observed for those using CHCA and 9-AA as matrices in different ionization modes, while less background interference for IVONSs:Sm ranged from m/z 150 to m/z 600, especially in negative-ion mode, further demonstrating the potential of IVONSs:Sm as a stable nano-matrix for LDI-MS analysis.

Furthermore, the study evaluated the performance of LDI-MS using IVONSs:Sm in contrast to previously reported nano-matrices such as graphene and metal oxide (e.g., cerium oxide (CeO_2_)), which have been recognized for their superiority in SALDI-MS analysis of LWM compounds (Figure S4a, b) [34, 37]. Figure 2c, d revealed that MS signals of the four fatty acids could be distinctly detectable as [M − H]^−^ using the three nano-matrices. However, the higher analyte signals for IVONSs:Sm suggested its superiority over graphene and CeO_2_ in detection sensitivity (Fig. 2e), which could be ascribed to a synergistic effect of stronger UV absorption, higher photothermal capability of IVONSs:Sm, and potentially effective interactions between IVONSs:Sm and surface LMW compounds. As shown in Figure S4c, UV-vis absorption spectra of the three nano-matrices with the same dispersing concentration were investigated. A stronger absorption of IVONSs:Sm than other nano-matrices at the operational wavelength (355 nm) made it possible to be utilized as a benign receptor of laser energy in the LDI-MS process. Additionally, the infrared thermographic images and photothermal heating curves of the three nano-matrices indicated that IVONSs:Sm had more advantageous photothermal conversion compared to the other two conventional nano-matrices, which made it effective to transfer the absorbed laser energy and enhance the ionization of analytes (Figure S5). With this in mind, it could be an optimal choice to analyze LMW compounds with IVONSs:Sm as a nano-matrix. Furthermore, Fig. 2e and Figure S6 suggested that the incorporation of Sm^3+^ into IVONSs could be synergistically contributive to the negative ionization process. Significantly, the MS spectrum with the same characteristic peaks of the four fatty acids but insufficient MS intensities was obtained by using IVONSs without samarium doping. This outcome demonstrated the superior feasibility of IVONSs:Sm over pure IVONSs in the analysis of fatty acids. Accordingly, the optimal dopant amount was investigated, and Fig. 2f indicated that the MS signal intensities of the four fatty acids reached their maximum at 5.0 mol % samarium, suggesting an optimal samarium molar ratio for IVONSs:Sm.

### Mechanistic basis of IVONSs:Sm-assisted LDI-MS

**Fig. 3:**
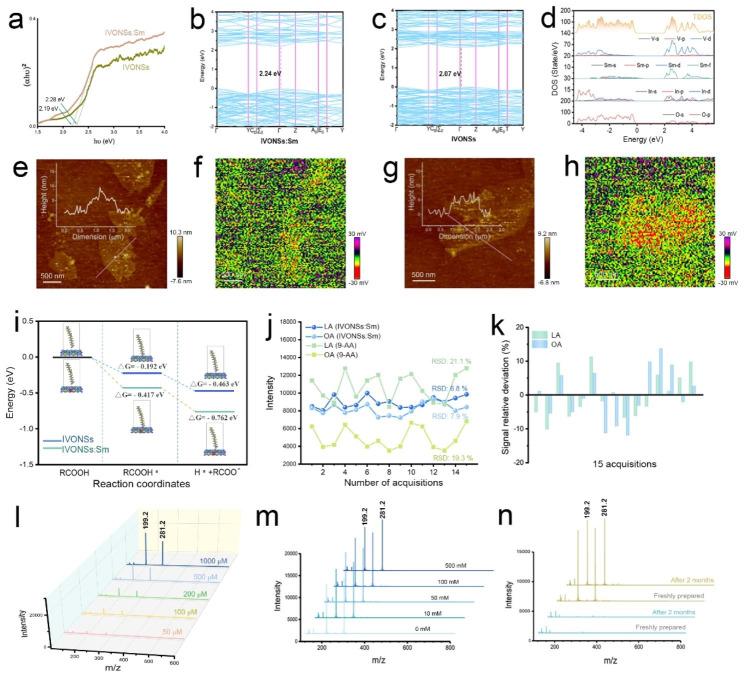
(**a**) Tauc plots of IVONSs:Sm and IVONSs. Energy band structure of (**b**) IVONSs and (**c**) IVONSs:Sm. (**d**) Calculated DOS of IVONSs:Sm. (**e**) AFM and (**f**) SPV images of IVONSs, and (**g**) AFM and (**h**) SPV images of IVONSs:Sm. (**i**) Calculated Gibbs free-energy diagram for the adsorption and dissociation process of fatty acid over IVONSs:Sm and IVONSs surfaces, respectively. (**j**) Reproducibility of MS signal intensities for LA and OA using different matrices. (**k**) Signal relative deviation of MS signal intensities using IVONSs:Sm. (**l**) IVONSs:Sm-assisted LDI-MS spectra of different concentrations of LA and OA in negative-ion mode. (**m**) IVONSs:Sm-assisted LDI-MS spectra of LA and OA (500 µM) in different concentrations of NaCl in negative-ion mode. (**n**) IVONSs:Sm-assisted LDI-MS spectra of a blank sample (green lines) and a mixture of LA and OA (yellow lines) with freshly prepared IVONSs:Sm and IVONSs:Sm after two months of preservation

While the precise LDI mechanism remains to be conclusively established, numerous studies have provided evidence supporting the photoexcitation and electronic transition mechanism induced by laser in the ionization process [38]. Hence, the enhancement of MS signals with IVONSs:Sm could also be elucidated by the electronic transition mechanism for the negative ionization of LMW molecules. As depicted in Fig. 2g (inset), the input laser energy could be transduced by generation of electron-hole pairs in IVONSs:Sm, where the excited electrons could be emitted from IVONSs:Sm and subsequently interact with LMW molecules, facilitating the negative ionization of the analyte [M − H]^−^. To validate this mechanistic basis, potassium thiocyanate was employed as a hole-scavenger to investigate the correlation between the generation of electron-hole pairs and the LDI process of LMW molecules [38]. Figure 2g demonstrated decreasing deprotonated ion peaks of of LA (m/z 199.2) and OA (m/z 281.2) with an increase in KSCN concentration (0, 5, 10, 20 µmol/mL), showing a distinct suppression of the MS signal of the two fatty acids in the presence of higher KSCN concentration, thus experimentally supporting the electronic transition mechanism in Fig. 2g [39]. Consequently, the electronic structure and optoelectronic properties of IVONSs:Sm should be examined. Figure 2h, i and Figure S7 presented the electron density analysis of IVONSs and IVONSs:Sm on the crystal face (100). In comparison to pure IVONSs (Figure S7), the distinct electron cloud overlapping in IVONSs:Sm confirmed the coexistence state of electrovalent bond and covalent bond in samarium-oxygen polarity linkages (Fig. 2h). Accordingly, the differential electron density diagram indicated that the electron density of O^2-^ adjacent to Sm^3+^ increased (Fig. 2i). Thus, the octahedron formed by Sm^3+^-O^2-^ bond caused the enhancement of the electrical dipole moment in the lattice, which was beneficial for the diffusion of the charge carrier. On the other hand, Figure S8a depicted the diffuse reflectance ultraviolet-visible spectra of IVONSs:Sm and pure IVONSs. Notably, the strong absorption bands ranging from 200 to 400 nm were mainly attributed to the charge transfer effect between V^5+^–O^2-^ and f-f transitions of Sm^3+^ (Figure S8b) [40, 41], implying that IVONSs:Sm predominantly absorbed laser power (e.g., 355 nm Nd:YAG laser) in the MALDI-MS instrument, thus allowing for the subsequent ionization process. According to the Tauc equation, the corresponding band gaps of IVONSs:Sm and pure IVONSs based on the optical absorption were calculated to be 2.19 and 2.28 eV, respectively (Fig. 3a). The lower band gap of IVONSs:Sm than pure IVONSs was further supported by density functional theory (DFT) simulation (Fig. 3b, c), where the DFT-derived band gap of pure IVONSs aligned with the experimental data. For IVONSs:Sm, the direct band gap of IVONSs:Sm was slightly smaller than the experimental value, as DFT simulation tends to underestimate the band gap [42]. The density of states (DOS) results of IVONSs indicated that the conduction band was predominantly composed of V 3d states (Figure S9a), while the V 3d states hybridized with the Sm 4f states, forming the conduction band of IVONSs:Sm (Fig. 3d). This suggested that the Sm 4f states would shift the conduction band towards the lower energy, thus reducing the band gap for IVONSs:Sm. The experimental and calculation results consistently demonstrated the decrease in the IVONSs band gap with Sm ion dopant. Importantly, the lower band gap of IVONSs:Sm promoted the electronic transition and charge carrier generation, which was conducive to the negative ionization of analytes as per the mechanism illustrated above.

In addition, Fig. 3e ~ h depicted atomic force microscope (AFM) and surface photovoltage (SPV) images of the two vanadate nanosheets, providing a spatial visualization of the generation and transport of charge carriers at the nanoscale. The AFM images of IVONSs:Sm and pure IVONSs, with similar lateral dimensions and thickness of approximately 5 nm, were presented in Fig. 3e and g. However, the corresponding SPV images, obtained by subtracting the potentials under dark conditions, revealed that IVONSs:Sm exhibited a more negative light-induced potential change of approximately 30 mV compared to pure IVONSs. This indicated a higher accumulation of electrons and greater mobility of charge carriers from the bulk phase to the surface of IVONSs:Sm, which facilitated energy transfer from the nanomaterial to LMW molecules, consequently increasing the efficiency of negative ionization in LDI-MS. To gain insight into the role of the nanomaterial IVONSs:Sm in the LDI process, we further investigated the thermodynamic profile of adsorption and dissociation of analytes on the two vanadate nanosheets using DFT simulations. As a representative LMW molecule, the electrostatic potential (ESP) of OA was initially calculated based on the ground state electron density (Figure S9b). The carboxyl group was found to be favorable for OA (RCOOH) to be adsorbed onto the vanadate nanosheets (RCOOH*) due to its maximum ESP. Consequently, the acidic hydrogen in the carboxyl terminal tended to transfer from OA to the surface lattice oxygen, liberating the negative ion of OA (RCOO^−^). Figure 3i demonstrated that the Gibbs free energies of each step were reduced on IVONSs:Sm relative to pure IVONSs, indicating that the LDI process on the surfaces of IVONSs:Sm was thermodynamically favorable. Taking all of the above into consideration, the improved performance of IVONSs:Sm in nanomaterial-assisted LDI could be elucidated as a synergistic effect of various factors, including optical absorption and charge carrier mobility.

### IVONSs:Sm-assisted LDI-MS analysis of fatty acids

Due to its superior performance, IVONSs:Sm was determined to be a highly effective nano-matrix for in situ detection of LMW compounds in authentic samples, such as fingerprints. A lifting process using double-sided conductive copper foil tape was performed to retrieve fingerprints from surfaces. The exceptional ability of IVONSs:Sm in negatively ionizing LMW compounds on the copper conductive tape was also verified (Figure S10). To assess the reproducibility of IVONSs:Sm-assisted LDI-MS in negative-ion mode, the mass spectrometry signal intensities of representative LA (saturated fatty acid) and OA (unsaturated fatty acid) from fifteen randomly selected positions within a single spot (diameter ~ 2.1 mm) of analytes spotted on the copper conductive tape were collected. As shown in Fig. 3j, relatively stable MS signals for LA and OA were observed, and a low relative standard deviation for each acquisition confirmed the high shot-to-shot reproducibility of the IVONSs:Sm-assisted LDI-MS approach (Fig. 3k). In comparison, the MS signal intensities for LA and OA showed significant fluctuations with an RSD of ~ 20% for each acquisition (Fig. 3j), indicating unsatisfactory repeatability when employing the traditional organic matrix 9-AA. The good shot-to-shot reproducibility was mainly attributed to the difference in the homogeneity of IVONSs:Sm and 9-AA deposited on the copper conductive tape. Figure S11 demonstrated the crystallization of IVONSs:Sm and two types of traditional organic matrices (CHCA and 9-AA) obtained in a MALDI-MS spectrometer, in which a stark difference of matrix distribution between IVONSs:Sm and other two organic matrices could be witnessed. It could be seen that IVONSs:Sm spread more uniformly after the solvent evaporation, instead of exhibiting the “sweet spot” effect of CHCA and 9-AA on the target surface. The good shot-to-shot reproducibility effectively solved the variability of signal intensity, providing assurance of reliable quantitative analysis of analytes using IVONSs:Sm. Fig. 3L demonstrated that the MS peaks of the two deprotonated fatty acids [M − H]^−^ gradually raised with the increasing analyte concentrations. The MS responses for LA at m/z 199.2 and OA at m/z 281.2 were proportional to the concentrations of the target analytes (Figure S13a), and the detection limit (LOD) investigations of LA and OA corroborated the sensitivity of the IVONSs:Sm-assisted LDI-MS approach (8.2 µM for LA and 11.6 µM for OA). As shown in Figure S12, a quantitative analysis of residual LA and OA in real fingerprint samples was further performed using high-performance liquid chromatography-electrospray ionization mass spectrometry (HPLC-ESI-MS). The residual quantities of LA and OA in a fingerprint sample were determined to be 0.36 and 2.72 µg, respectively. Taking the LODs and added volume (1 µL) of fatty acid standard solutions into account, the proposed IVONSs:Sm-assisted LDI-MS approach was capable of detecting 1.6 × 10^− 3^ µg of LA and 3.3 × 10^− 3^ µg of OA in real samples, which were substantially lower than those detected by HPLC-ESI-MS. Therefore, the quantitative results supported that IVONSs:Sm-assisted LDI-MS could satisfy the analytical demand of fatty acid levels in fingerprints. Given that a high salt concentration might suppress the ionization process of the analyte, the salt tolerance was evaluated by adding different concentrations of NaCl (0 ~ 500 mM) in IVONSs:Sm-assisted LDI-MS detection of two representative fatty acids. Figure 3m showed that the addition of 10–500 mM NaCl slightly decreased the signal intensities of LA and OA, confirming a good tolerance of the nano-matrix IVONSs:Sm. Additionally, it was noted that the characteristic MS peaks of the LA and OA mixture using IVONSs:Sm with a two-month storage matched those using freshly prepared IVONSs:Sm (Fig. 3n), and there was insignificant divergence in the pattern and intensity of the MS peaks after multiple laser shots (Figure S13b, c). Hence, the stability of IVONSs:Sm, including the photostability of analytes during IVONSs:Sm-assisted LDI-MS analysis, could be experimentally ascertained.

### IVONSs:Sm-assisted LDI-MS imaging of fingerprints

**Fig. 4:**
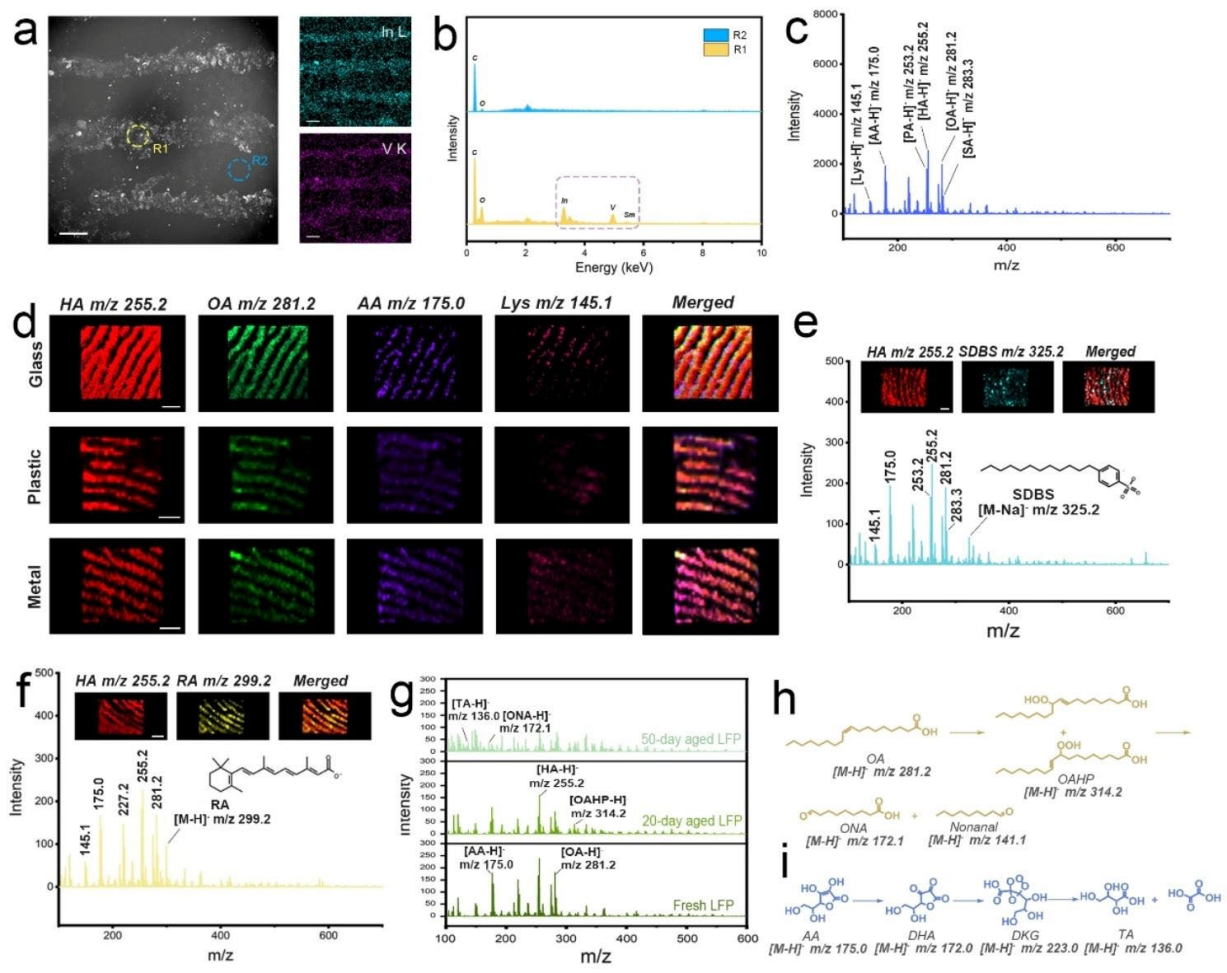
(**a**) SEM image of a fingerprint sprayed with IVONSs:Sm and corresponding EDS mapping of In and V elements. (**b**) EDS spectra of circular regions (ridge and valley in a fingerprint) in the SEM image. (**c**) IVONSs:Sm-assisted LDI-MS spectra of a fingerprint in negative-ion mode. (**d**) MS images of the fingerprints collected from different substrates in negative-ion mode. IVONSs:Sm-assisted LDI-MS analysis for (**e**) SDBS and (**f**) RA on fingerprints. (**g**) IVONSs:Sm-assisted LDI-MS spectra of fresh and aged fingerprints in negative-ion mode. Proposed oxidation pathway of (**h**) OA and (**i**) AA during fingerprint storage. Scale bars in (**d**)~(**f**) represent 1 mm

Based on the aforementioned study, the applicability of IVONSs:Sm for detecting LMW compounds adhered to fingerprints was preliminarily investigated. IVONSs:Sm were dispersed in bromoethane with a low boiling point and then sprayed on the fingerprint section through an atomizer. Following the deposition of IVONSs:Sm, the fingerprint was lifted using copper conductive tape. Scanning electron microscopy (SEM) images and results from EDS analysis confirmed the concentration of IVONSs:Sm on the ridges of the fingerprint after the disperse medium evaporation (Fig. 4a, b). To collect IVONSs:Sm-assisted LDI-MS spectra of the fingerprints, the copper conductive tape containing the extracted fingerprint was affixed to an indium tin oxide-coated glass slide for IVONSs:Sm-assisted LDI-MS analysis. Figure S14a presented the positive MS profile obtained from the extracted fingerprint, which showed the MS spectrum of an endogenous mixture containing PA, hexadecanoic acid (HA), OA, and stearic acid (SA). The abundance of MS peaks confirmed that the chemical information of the fingerprint has been transferred onto the substrate of the copper conductive tape. Apart from the fatty acids, other ion peaks originated from other endogenous compounds were also recorded in the range m/z 100–700. For example, the peaks detected at m/z 147.1 and 177.1 were assigned to the [M + H]^+^ ion of lysine (Lys) and ascorbic acid (AA), respectively. In contrast, the MS spectrum of the extracted fingerprint obtained in negative-ion mode exhibited a relatively concise signal with long-term stability in high vacuum (Fig. 4c and Figure S14b ~ d). Unlike the multiple peaks for one analyte under positive-ion mode, all the fatty acids were clearly detected as the only deprotonated [M − H]^−^ ions with the assistance of IVONSs:Sm. Additionally, intense ions in the fingerprint were putatively identified via MALDI LIFT-TOF/TOF MS (Figure S15, S16, and Table S3). Furthermore, considering the coexistent lipids in fingerprints, as well as the potential ester bond cleavage in the case of IVONSs:Sm, a controlled experiment was performed and indicated a relatively stable MS signal of two model fatty acids in the existence of two typical lipids (glycerol trimyristate and glycerol tripalmitate), which excluded the interference of lipids on subsequent fatty acid analysis (Figure S17). All the results suggested the preponderance and feasibility of negative-ion IVONSs:Sm-assisted LDI-MS for spatial molecular profiling in fingerprints.

Since IVONSs:Sm allowed for the MS analysis of chemical species contained within the fingerprint, IVONSs:Sm-assisted LDI-MS imaging of the fingerprint could be generated from the spatial distribution of the MS signals of selected deprotonated analytes. Considering the spraying conditions of IVONSs:Sm could affect the loading amount and homogeneous coverage of the nano-matrix on the fingerprint, critical experimental parameters (including dispersing concentration of IVONSs:Sm and spraying time) were optimized. For the sake of simplicity, HA was taken as the representative fatty acid for IVONSs:Sm-assisted LDI-MS analysis in negative-ion mode. As shown in Figure S18a, the vanadium content on the substrate of the copper conductive tape was determined by inductively coupled plasma mass spectrometry (ICP-MS), which implied that the loading amount of IVONSs:Sm on the fingerprint gradually increased with the increase of IVONSs:Sm concentration (Figure S18b). As a consequence, the MS signal of HA at m/z 255.2 went up with the dispersing concentration of IVONSs:Sm. Likewise, the spraying time was optimized, and the results were presented in Figure S18c. The incremental vanadium content and the MS signal with the spraying time of IVONSs:Sm suspension further confirmed that adequate IVONSs:Sm deposition was beneficial to the enhancement of the MS signal. Aside from the evaluation of spraying conditions in light of MS signal intensity of the analyte, the performance of IVONSs:Sm-assisted LDI-MS imaging could also depend on parameters like the dispersing concentration of IVONSs:Sm and spraying time. It could be found that the MS images with higher image clarity and contrast were obtained with the increasing concentration of IVONSs:Sm (Figure S19a ~ c). Figure S19d ~ f indicated that the MS images of the fingerprint gradually blurred when the spraying time increased to 60 s, suggesting that the prolonged deposition procedure of IVONSs:Sm resulted in an undesirable MS image without the original minutiae of the fingerprint pattern. This phenomenon might be ascribed to the factor that extended and continuous exposure of sprayed bromoethane droplets could thoroughly wet the fingerprint area, causing the delocalization and diffusion of hydrophobic fatty acids, thus the corresponding MS image of the fingerprint was obtained with poor spatial resolution. In this case, the proper spraying time enabled a well-defined MS image in fingerprint analysis. Based on the above results, the optimal parameters of dispersing concentration (5 mg/mL) and spraying time (30 s) of IVONSs:Sm suspension for the matrix deposition could be settled.

In order to investigate the feasibility of the suggested IVONSs:Sm-assisted LDI-MS method for fingerprint analysis, the fingerprints left on different material surfaces were analyzed via IVONSs:Sm-assisted LDI-MS imaging in negative-ion mode. As shown in Fig. 4d, the fingerprint morphological features could be clearly perceived, and the MS images could also provide the spatial distribution of characteristic LMW compounds detected at m/z 145.1 (Lys), 175.0 (AA), 255.2 (HA), and 281.2 (OA). Importantly, there was no definite difference among these three material surfaces in imaging quality. These results prove the capability of IVONSs:Sm-assisted LDI-MS imaging to allow molecular-level fingerprint recognition on different material surfaces. Figure S20 demonstrated that the imaging effect using IVONSs:Sm compared favorably with those of graphene and CeO_2_. Compared to graphene and CeO_2_ (Table S4), the highest signal intensity and the largest number of detectable endogenous LMW compounds in fingerprints were obtained using IVONSs:Sm (Figure S20a). As seen in Figure S20b, fingerprint morphological features and spatial distribution of two representative LMW compounds (OA and Lys) could be clearly visualized, while relatively lower MS intensities of LMW compounds from the nano-matrix graphene affected the applied performance of MS imaging for the fingerprint (Figure S20c). Moreover, a lower contrast between ridges and valleys of the fingerprint was observed from the MS images using the nano-matrix CeO_2_ (Figure S20d). This phenomenon might be mainly ascribed to the low hydrophobicity of the nano-matrix CeO_2_ (Figure S21), which led to an uneven dispersion of CeO_2_ in a weak-polar suspending medium and hence inhomogeneous coverage spotted on the fingerprint. In addition, more hydrophilic CeO_2_ could also reduce the performance of LDI-MS imaging via attenuated interaction between the nano-matrix and hydrophobic LMW compounds (e.g., fatty acids). All of this suggests that IVONSs:Sm with appropriate hydrophobicity and high LDI capability could surpass nano-matrices graphene and CeO_2_ in the imaging quality of LMW compounds in fingerprints. Moreover, given the analysis of exogenous compounds on the fingerprint, such as individual skincare products or drug residues, is of great value to reconstruct a lifestyle profile of the fingerprint donor, the exogenous fingerprints were prepared after using a liquid soap or acne cream, then analyzed via IVONSs:Sm-assisted LDI-MS imaging. Figure 4e exhibited a negative-ion mode MS spectrum and the derived MS images of the two representative compounds, namely HA (endogenous) and sodium dodecyl benzene sulfonate (SDBS, exogenous). SDBS is a widely used surfactant in personal care products, and the related [M − Na]^−^ ion of SDBS was detected at m/z 325.2. The existence and spatial distribution of this exogenous ingredient could be verified by the MS image, which overlapped well with the fingerprint pattern derived from the negative-ion at m/z 255.2 of HA. Similarly, fingerprint chemical images of HA and the key pharmaceutical component (retinoic acid, RA) in acne cream were extracted from the MS spectrum to display the fingerprint pattern. Figure 4f confirmed that the fingerprint ridge details could be observed using the intensity map of [M − H]^−^ ion of HA (m/z 255.2) or RA (m/z 299.2). To further evaluate the detectability of exogenous LMW compounds on the fingerprint, fingerprint samples with different content levels of SDBS, in which SDBS was selected as model compounds of exogenous analyte, were collected after washing hands with the diluted lotions of gradient dilution ratios (Figure S22a). It was found that SDBS could be detected with a signal-to-noise ratio (S/N) of 5.1 even at a dilution ratio of 1/10. Besides, the LODs of SDBS and RA were calculated to be 3.6 ng and 12.9 ng, respectively. Consequently, the IVONSs:Sm-assisted LDI-MS method demonstrated gratifying sensitivity and great prospects in monitoring exogenous compounds on the fingerprint, especially in the field of forensic science. This capability to sensitively determine fingerprint morphology and chemical information would greatly make the fingerprint spotted at the scene of a crime valuable, particularly for those fingerprints that were not included in the existing fingerprint database.

**Fig. 5:**
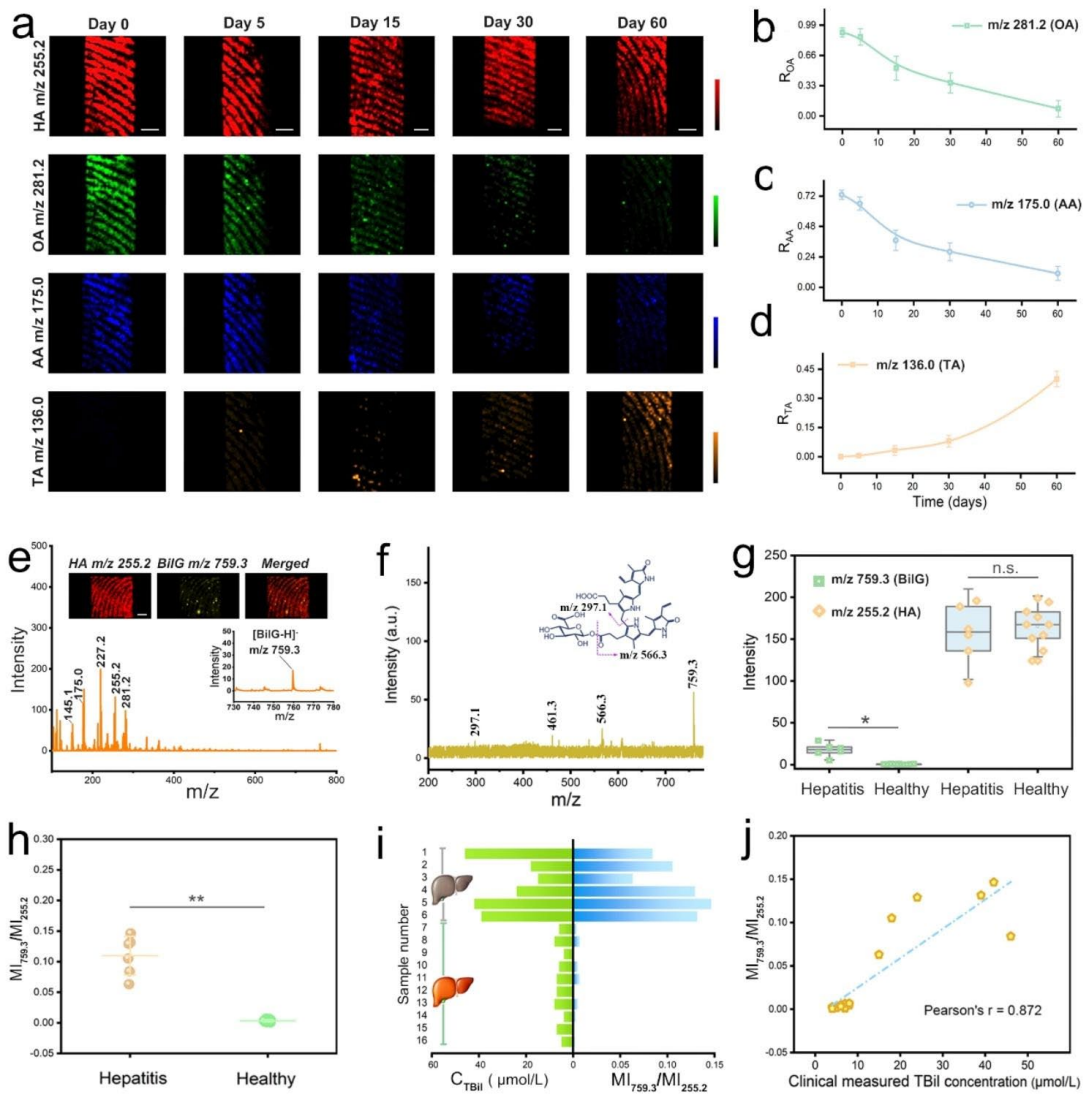
(**a**) IVONSs:Sm-assisted LDI-MS images of fresh and aged fingerprints in negative-ion mode, and time-dependent MS intensity ratios of (**b**) OA, (**c**) AA, and (**d**) TA. (**e**) Representative IVONSs:Sm-assisted LDI-MS spectra of fingerprints from a hepatitis patient. The inset shows the corresponding spatial distribution of HA and BilG. (**f**) The MALDI LIFT-TOF/TOF MS/MS spectrum of BilG obtained from a fingerprint sample in negative-ion mode. (**g**) Box plot of MS signal intensity at m/z 255.2 and 759.3 between healthy volunteers and hepatitis patients (* P < 0.05). (**h**) Scattered dot plots of the calculated MS signal intensity ratio (MI_759.3_/MI_255.2_) between healthy volunteers and hepatitis patients (* P < 0.01). (**i**) Comparison between MI_759.3_/MI_255.2_ and clinically measured TBil concentration from 6 hepatitis patients (No. 1 ~ 6) and 10 healthy volunteers (No. 7 ~ 16). (**j**) Correlation between MI_759.3_/MI_255.2_ and clinically measured TBil concentration from 6 hepatitis patients and 10 healthy volunteers. Scale bars in (**a**) and (**e**) represent 1 mm

On the other side, we observed that the age of the fingerprint was another evident piece of information to narrow down the persons of interest. Stimulated by the good performance shown above, an attempt was undertaken to assess the age of the fingerprint using the IVONSs:Sm-assisted LDI-MS tool. For the sake of accuracy, all the fingerprint samples were stored at 30 °C and 60% relative humidity (RH). Figure 4g presented MS spectra of the representative fresh and aged fingerprints in the negative-ion mode. The unsaturated OA was found to undergo peroxidization reaction, resulting in a signal decrease at m/z 281.2, while the MS signals of saturated HA in fingerprints were relatively stable over time. At the same time, two additional peaks related to the degradation of OA appeared with prolonged fingerprint age, which were identified as oleic acid hydroperoxide (OAHP, m/z 314.2) and 9-oxo-nonanoic acid (ONA, m/z 172.1). The peroxidization mechanism of OA based on the former studies was given in Fig. 4h [43]. Namely, OA undergoes a radical-mediated autoxidation in ambient air, forming isomerization products of OAHP, secondary oxidation products like ONA and nonanal are derived from the cleavage of OAHP. Likewise, the decrease of the oxidizable AA and the new peak emerged at m/z 136.0, which was assigned to threonic acid (TA), confirmed the oxidative degradation pathway of AA. The widely accepted mechanism of AA oxidation suggested the generation of a series of active intermediates (e.g. dehydroascorbic acid (DHA), diketogulonic acid (DKG)) and threonic acid (TA) [42, 44]. That is, DHA, an oxidized form of AA, is then hydrolyzed to DKG, the intermediate DKG is further decomposed to TA after the rearrangement reaction (Fig. 4i). The corresponding MS images were compelling support for these trends of OA and AA over time. The MS image of HA spatial pattern remained clearly visible and practically invariable on the fingerprint ridges over time (Fig. 5a). In contrast, the fingerprint patterns gradually faded for OA and AA from day 0 to day 60. The MS image of TA was absent during the initial period of fingerprint aging but became much clearer with time going on. These results gave a visual demonstration of the time-dependent changes of the representative LMW compounds in fingerprints. Importantly, quantitative analysis of the changes in the LMW compounds implied the possibility of tracking the age of fingerprints. To reduce systematic error, the MS signals were normalized by calculating the intensity ratios (R_OA_, R_AA_, and R_TA_) using the following equations: R_OA_ = I_OA_/I_HA_, R_AA_ = I_AA_/I_HA_, R_TA_ = I_TA_/I_HA_, where I_OA_, I_AA_, and I_TA_ represented the MS signal intensities of OA at m/z 255.2, AA at m/z 175.1, and TA at m/z 136.0, respectively, and IHA stood for the HA intensity at m/z 281.2 with a non-significant change. Hence, Fig. 5bd illustrate the curves of R_OA_, R_AA_, and R_TA_ over a two-month period of fingerprint aging, respectively. The decrease of R_OA_ (R_AA_) and the increase of R_TA_ were consistent with the differences in the MS images in Fig. 5a. Similarly, Figure S22b ~ d provided a consistent trend of the calculated MS intensity ratios (R_OA_, R_AA_, and R_TA_) over time compared to Fig. 5b ~ d. The insignificant difference among all the groups suggested a tiny influence of ambient fluctuations on the curves of R_OA_, R_AA_, and R_TA_, confirming the validity of the means of establishing fingerprint age. Moreover, for a simulation experiment for fingerprint age determination, ten simulated fingerprint specimens with different aging times were analyzed via the IVONSs:Sm-assisted LDI-MS approach. Semi-quantitative evaluation of fingerprint age was performed based on the time-dependent MS intensity ratios (R_OA_, R_AA_, and R_TA_). Inference results for fingerprint aging time were derived by adopting the average aging time of the three analytes. Comparisons between the true and predicted results were visualized by a heat map, and the color of the squares denoted the gradation of fingerprint age (Figure S23a). Verification with low prediction error proved the practicality of the IVONSs:Sm-assisted LDI-MS tool for determining the aging time of unknown fingerprint samples.

We subsequently sought to extend the application of the IVONSs:Sm-assisted LDI-MS method to monitor biomarkers excreted from finger sweat glands. It was found that elevated levels of free bilirubin (bilirubin glucuronide, BilG) in the biofluids is related to various hepatopathies [45]. Hence, rapidly monitoring the level of BilG in a label-free manner is of great importance for early diagnosis (Figure S23b). With its reliable, highly sensitive, and label-free features, IVONSs:Sm-assisted LDI-MS is particularly well-suited for trace BilG detection. The typical MS spectrum of the sweat fingerprint sample from acute hepatitis patients displayed the [M − H]^−^ peak of BilG at m/z 759.3, which was putatively identified via MALDI LIFT-TOF/TOF MS (Fig. 5e, f). Additionally, the MS images exhibited a sweat pore-centered spatial distribution of BilG in the fingerprint ridge area (inset, Fig. 5e). For the sweat fingerprint sample from a healthy donor, a MS spectrum without a BilG signal was observed (Figure S23c). To further evaluate the feasibility of the proposed IVONSs:Sm-assisted LDI-MS tool in medical analysis, BilG levels in fingerprints from 10 healthy donors and 6 hepatitis patients were analyzed. Figure S24 enumerated the negative-ion mode MS spectra of fingerprints from the hepatitis patient group, and higher MS intensity at m/z 759.3 than the healthy group pointed out the increase of BilG concentration in the sweat of hepatitis patients (Fig. 5g and Figure S25). By using the MS signal of HA (m/z 255.2) as an internal standard, this discrepancy could be more clearly discriminated via a ratiometric parameter (MI_759.3_/MI_255.2_), where MI_759.3_ and MI_255.2_ represented the MS signal intensity at m/z 759.3 and 255.2, respectively (Fig. 5h). Furthermore, the serum total bilirubin (TBil) level of the 16 participants, which was widely used in serodiagnosis, was determined by the clinically bilirubin oxidase method. Figure 5i showed a good consistency between the parameter MI_759.3_/MI_255.2_ and TBil concentration in identifying hepatitis patients, which was further corroborated by Pearson correlation analysis (r = 0.872) in Fig. 5j. Due to the superiorities of the label-free manner, non-invasive sampling, and quick response, the proposed method possesses outstanding advantages over many bilirubin assay techniques [46, 47].

## Conclusion

In brief, the initial synthesis of IVONSs:Sm nanosheets was accomplished, and subsequently, these nanosheets were incorporated into a negative-ion SALDI-MS system as an appropriate nano-matrix for the detection of LMW compounds. The IVONSs:Sm exhibited distinct advantages in the interpretation and reproducibility of MS spectra when compared to conventional organic matrices. Furthermore, the application of the IVONSs:Sm-assisted LDI-MS method to fingerprint analysis demonstrated a multitude of advantageous characteristics. First, the successful recognition of fingerprint ridge patterns was accomplished by employing a method that minimizes background interference and amplifies signal intensity. Second, a thorough examination of fingerprints, taking into account the amalgamation of nuanced molecular-level variations and disparities throughout time, proved to be sufficient in yielding vital biometric data and forensic proof. Furthermore, the utilization of copper conductive tape facilitated the extraction of fingerprints, hence expanding the method’s potential for various non-conductive materials. Additionally, the label-free and non-invasive characteristics of the IVONSs:Sm-assisted LDI-MS platform render it exceptionally well-suited for the advancement of preventive and diagnostic healthcare applications. Hence, a revolutionary framework for multidimensional fingerprint analysis was introduced in this study, which not only introduced an innovative nano-matrix for negative-ion LDI-MS but also facilitated the monitoring of specific targets in biosamples.

### Electronic supplementary material

Below is the link to the electronic supplementary material.

Supplementary Material 1: Experimental section and additional data (Figure S1–S25, Table S1–S4) associated with this article can be found in the online version

## Data Availability

Data will be made available on request.
