# Correction: Temporally and spatially resolved molecular profiling in fingerprint analysis using indium vanadate nanosheets-assisted laser desorption ionization mass spectrometry

**DOI:** 10.1186/s12951-023-02292-5

**Published:** 2024-01-30

**Authors:** Yanli Zhu, Jikai Wang, Chengxiao Fu, Shuangquan Liu, Pragati Awasthi, Pengfei Zeng, Danjun Chen, Yiyang Sun, Ziyi Mo, Hailing Liu

**Affiliations:** 1 Hunan Province Cooperative Innovation Center for Molecular Target New Drug Study, Institute of Pharmacy & Pharmacology, Hengyang Medical School, University of South China, Hengyang, Hunan 421001 P. R. China; 2 School of Resources and Environment, Hunan University of Technology and Business, Changsha, Hunan 410205 P. R. China; 3 The First Affiliated Hospital, Department of Clinical Laboratory, Department of Pharmacy, Hengyang Clinical Pharmacology Research Center, Hengyang Medical School, University of South China, Hengyang, Hunan 421001 P. R. China; 4 State Key Laboratory of Silicon Materials & School of Materials Science and Engineering, Zhejiang University, Hangzhou, Zhejiang 310058 P. R. China; 5 Department of Respiratory and Critical Care Medicine, Renmin Hospital of Wuhan University, Wuhan, Hubei 430060 P. R. China

**Correction: Journal of Nanobiotechnology (2023)21:475**

10.1186/s12951-023-02239-w

Following publication of the original article [1], the authors identified an error in affiliation 1 due to a typesetting mistake. The affiliation was incorrectly given as “school of Resources and Environment, Hunan University of Technology and Business, Changsha, Hunan 410205, P. R. China”, but should have been “Hunan Province Cooperative Innovation Center for Molecular Target New Drug Study, Institute of Pharmacy & Pharmacology, Hengyang Medical School, University of South China, Hengyang 421001, Hunan, P.R. China”.

This error is corrected in the affiliations list below and the original article [1] has been revised. The publisher apologises to the authors and readers for the inconvenience caused by this error.
