# Supplementary material for: Temporally and spatially resolved molecular profiling in fingerprint analysis using indium vanadate nanosheets-assisted laser desorption ionization mass spectrometry
Source: J Nanobiotechnology. 2023 Dec 10;21:475. doi: 10.1186/s12951-023-02239-w (PMC10710729; doi:10.1186/s12951-023-02239-w)
Supplement: Supplementary file 1 — Supplementary Material 1: Experimental section and additional data (Figure S1–S25, Table S1–S4) associated with this article can be found in the online version [file 12951_2023_2239_MOESM1_ESM.docx]

Supporting Information:

**Temporally and Spatially Resolved Molecular Profiling in Fingerprint Analysis Using Indium Vanadate Nanosheets-Assisted Laser Desorption Ionization Mass Spectrometry**

Yanli Zhu ^2^, Jikai Wang ^1 *^, Chengxiao Fu ^3^, Shuangquan Liu ^3^, Pragati Awasthi ^4^, Pengfei Zeng ^1^, Danjun Chen ^3^, Yiyang Sun ^1^, Ziyi Mo ^1^, and Hailing Liu ^5^

^1^ Hunan Province Cooperative Innovation Center for Molecular Target New Drug Study, Institute of Pharmacy & Pharmacology, Hengyang Medical School, University of South China, Hengyang, Hunan, 421001, P. R. China.

^2^ School of Resources and Environment, Hunan University of Technology and Business, Changsha, 410205, Hunan, P. R. China.

^3^ The First Affiliated Hospital, Department of Clinical Laboratory, Department of Pharmacy, Hengyang Clinical Pharmacology Research Center, Hengyang Medical School, University of South China, Hengyang, 421001, Hunan, P. R. China.

^4^ State Key Laboratory of Silicon Materials & School of Materials Science and Engineering, Zhejiang University, Hangzhou, 310058, Zhejiang, P. R. China.

^5^ Department of Respiratory and Critical Care Medicine, Renmin Hospital of Wuhan University, Wuhan, 430060, Hubei, P. R. China.

* E-mail: [jkwang@hnu.edu.cn](mailto:jkwang@hnu.edu.cn) (J.K. Wang, corresponding author)

**Table of Contents**

**Experimental Section**

Computational Details

Fingerprint Samples Preparation and Analysis

HPLC-ESI-MS Equipment and Conditions for Fatty Acids Analysis

ICP-MS Measurement

Statistical Analysis

**Supplementary Data**

**Figure S1.** Elemental analysis of IVONSs:Sm

**Figure S2.** MS spectra of fatty acids using different matrices in positive-ion mode

**Figure S3.** MS spectra of blank matrices without analyte in different ionization modes

**Figure S4.** TEM images and UV-vis absorption spectra of different nano-matrices

**Figure S5.** Photothermal effect of the nano-matrices exposed to 405 nm laser irradiation

**Figure S6.** MS analysis of fatty acids using control matrices

**Figure S7.** Electron density profile of pure IVONSs

**Figure S8.** Optical absorption of the vanadate nano-matrices and dopants

**Figure S9.** Results of theoretical calculation of the vanadate nano-matrices and OA molecule

**Figure S10.** Evaluation of IVONSs:Sm-assisted LDI-MS method on copper conductive tape

**Figure S11.** Photographs of dried samples spotted with different matrices

**Figure S12.** Quantitative analysis of fatty acid residues in fingerprint sample

**Figure S13.** Feasibility and suitability of IVONSs:Sm for fatty acids detection

**Figure S14.** Stability of the IVONSs:Sm-assisted LDI-MS method for fingerprint analysis

**Figure S15.** Identification of endogenous LMW compounds detected from fingerprint samples

**Figure S16.** Possible Fragmentation Regularities of some endogenous LMW compounds

**Figure S17.** MS spectra of mixture of fatty acids and glycerides in negative-ion mode

**Figure S18.** Optimization of the IVONSs:Sm-assisted LDI-MS method for fingerprint analysis

**Figure S19.** MS images of fingerprints under different IVONSs:Sm spraying condition

**Figure S20.** Comparison of the three nano-matrices in MS imaging of fingerprint samples

**Figure S21.** Aqueous contact angle measurements of the nano-matrices

**Figure S22.** Time-dependent MS intensity ratio under different conditions

**Figure S23.** BilG detection in sweat fingerprint using IVONSs:Sm-assisted LDI-MS tool

**Figure S24.** Negative-ion mode MS spectra of fingerprint samples from healthy group

**Figure S25.** Negative-ion mode MS spectra of fingerprint samples from hepatitis group

**Supplementary Table**

**Table S1.** The operating parameters of ICP-MS experiments

**Table S2.** MS analysis result of fatty acids using CHCA, 9-AA, or IVONSs:Sm

**Table S3.** Putative identification of components from fingerprint samples

**Table S4.** Comparison between different nano-matrices used for fingerprint LDI-MS analysis

Experimental Section

**Computational Details.** First-principles calculations were performed using the Vienna ab initio simulation package (VASP).^[1,2]^ The generalized gradient approximation (GGA) of Perdew-Burke-Ernzerhof (PBE) was used to describe the exchange-correlation functional. The cut-off energy for the plane wave basis was set to 400 eV, and a 2 × 2 × 2 Monkhorst–Pack k grid was used for sampling the Brillouin zones during the structure calculations. Meanwhile, a 2 × 2 × 1 supercell structure was used to calculate the model. All the structures were fully relaxed (atomic position) up to 10-5 eV/Å force minimization and a maximum force of 0.01 eV/Å. The pseudopotential valence electrons of each atom used in the calculation are O (2s^2^2p^4^), V (3p^6^3d^4^4s^1^), In (5s^2^5p^1^), Sm (4f^6^5s^2^5p^6^6s^2^), respectively. The InVO_4_ (100) slab were modeled and a vacuum space exceeding 20 Å was employed to prevent interactions between two periodic units. The Grimme's DFT-D3 scheme of dispersion correction was adopted to describe the van der Waals (vdW) interactions in these systems. In all calculations, the Gibbs free energy was calculated as:

ΔG = Δ_E_+ΔG_correction_ (1)

ΔG_correction_ = E_ZPE_ +U(T)+TS(T)+PV (2)

Δ_E_ = E_final_ - E_initial_ (3)

where E_final_ and E_initial_ represented the final state energy and initial state energy, respectively. E_ZPE_, U(T) + PV, TS(T) stood for the zero-point energy, the enthalpic temperature correction, and the entropy corrections. ΔG_correction_ were calculated by VASPKIT.^[3,4]^ The color mapped isosurface graph of electrostatic potential (ESP) of OA was simulated using the DMol3 module of Materials Studio package (2016), the functionals used was BLYP of general gradient approximation (GGA), unless otherwise specified the default settings of the program were used.

**HPLC-ESI-MS Experimental Conditions for Fatty Acids Determination in Fingerprints.** The analysis of fatty acids in fingerprint samples was conducted using the 1200LC Series (Agilent Technologies, USA) coupled to the 6460 triple quadrupole mass spectrometer (Agilent Technologies, USA). Liquid chromatography separations were carried out on an XTerra MS C18 column (2.1 mm × 150 mm, 3.5 μm, Waters). The column oven and auto-sampler were set at temperatures of 40 °C and 4 °C, respectively. The flow rate of the mobile phase was 0.3 mL/min, and the injection volume was 5 μL. The mobile phase for the fatty acids assay comprised 5 mmol/L ammonium acetate (solvent A) and acetonitrile with 5 mmol/L ammonium acetate (solvent B). A 40-minute lipid elution gradient for the separation of fatty acids in the methanol extraction of fingerprints was performed as follows: for the first 5 minutes, the solvent composition was set at 40 % solvent A and 60 % solvent B, followed by a linear gradient to 20 % solvent A and 80 % solvent B for 3 minutes and maintained for 8 minutes. Then, the ratio of solvent B was decreased to 10 % in 2 minutes and held for the next 10 minutes. Finally, the mobile phase was changed from 10 % to 40 % solvent B linearly for 3 minutes and maintained at 40% solvent B isostatically for the last 9 minutes. The mass spectrometer was operated in negative-ion mode using an electrospray ionization (ESI) source. The typical operating source conditions were optimized with a capillary voltage of 2.0 kV, nozzle voltage of 500 V, and a nitrogen drying gas set at a flow rate of 800 L/min with a temperature of 350 °C. Acquisition was performed in single ion monitoring (SIM), and the [M-H]^-^ ion intensity of fatty acids was used for quantitative determination. Stock solutions of LA and OA were prepared by dissolving two fatty acid standards in methanol and stored at -20 °C. A working standard solution was prepared from the stock solutions by appropriate dilution in methanol. For the quantitative analysis, calibration curves for LA and OA were prepared in the range of 0.1~50 ng/µL. For the extraction of fatty acids, 200 µL of dichloromethane was added onto the fingerprint sample on a glass slide and incubated for 10 minutes. The extracting solution was collected into a clean microcentrifuge tube. This extracting step was repeated twice, and the merged dichloromethane solution containing fatty acids was dried under N_2_ and then reconstituted with 200 μL of methanol to obtain the fingerprint extraction for HPLC-ESI-MS analysis.

**ICP-MS Measurement.** The vanadium standard stock solution (500 mg/L, in 1 % HNO_3_, Aladdin Reagent, Co., Ltd.) was diluted with HNO_3_ (2 %, v/v) to prepare a series of working solutions. For IVONS:Sm loaded conductive tape sample preparation, the copper foil tape (~25 mg) was immersed in 5 mL HNO_3_ (35 %) and 1 mL H_2_O_2_ (30 %) for 2 h, and digested in a microwave according to the preset heating program. Afterward, the cooled solution was diluted to 100 mL with deionized water, then 2 mL of the diluted solution was further diluted by 50 times and finally subjected to ICP-MS for analysis. Similarly, the original copper foil tape was treated following the above procedure and served as a blank sample. For each sample, its mass spectral intensity of ^51^V was measured by an ICP-MS instrument equipped with an alumina sample injector tube, quartz torch, and micro-concentric nebulizer. All measurements were performed using instrumental software. The instrumental parameters of the ICP-MS instrument were listed in Table S1. Sample and instrumental-induced variations were compensated using internal standardization, namely choosing ^43^Sc as an internal standard and found to be negligible. Each sample was analyzed in three replications. The elemental content of the vanadium (C_V_) was calculated based on the following formulas: L_V_=(C_V_×5)/m. Where L_V_ (μg/mg) represents the loading amount of vanadium element on a unit mass of copper foil tape sample, C_V_ (μg/L) stands for the vanadium concentration of the test solution, and m is the precise weight of the copper foil tape sample.

**Table S1.** The operating parameters of ICP-MS experiments.

| **ICP-MS** | **Parameters** | |
| --- | --- | --- |
| ICP RF power | | 1300 W |
| Reflected power  Plasma gas flow | | 2 W  13 L/min |
| Auxiliary gas flow | | 0.76 L/min |
| Nebulizer gas flow | | 0.84 L/min |
| Isotope monitored | | ^51^V |
| Dwell time | | 30 ms |
| Sampling pump rate | | 3.0 mL/min |
| Sampling depth  Quadrupole bias | | 3.9 mm  -15.0 to 9.0 V |

**Ethics and Clinical Sample Analysis.** Collection and analysis of fingerprints specimens and clinical samples were in accordance with the protocols approved by the Biology and Medical Ethics Committee of University of South China, and the Medical Ethics Committee of the First Affiliated Hospital of University of South China. This study was also registered and documented in the Chinese Clinical Trial Registry (No. ChiCTR2200058628). All of serum samples from healthy donors and acute hepatitis patients were collected and analyzed in the First Affiliated Hospital of University of South China, the level of total bilirubin (TBil) was determined using automatic biochemical analyzer (Roche Modular PPI) based on the bilirubin oxidase method.

**Statistical Analysis.** All the experiments were repeated at least three times. The statistical data result was given as mean value ± standard deviation, and the statistical data was processed with SPSS software. Significant difference was determined using a Student’s t-test, and differences were set at P<0.01 (**), P<0.05 (*).

Supplementary Data

**
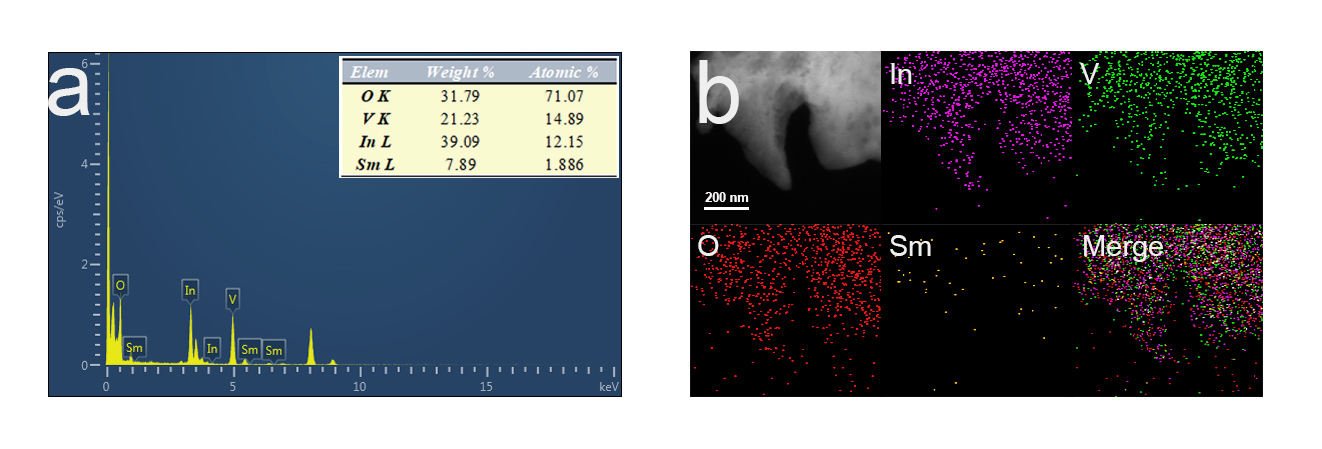
**

**Figure S1.** (a) EDX analysis and (b) elemental mapping for In, V, O, and Sm of IVONSs:Sm.


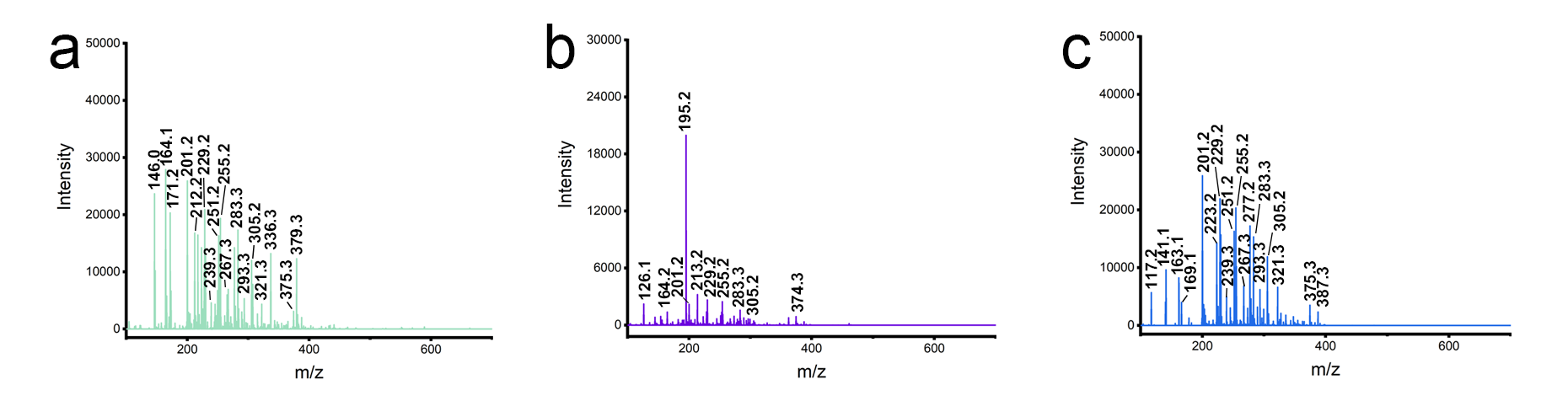


**Figure S2.** MS spectra of four fatty acids with (a) CHCA, (b) 9-AA, (c) IVONSs:Sm in positive-ion mode.


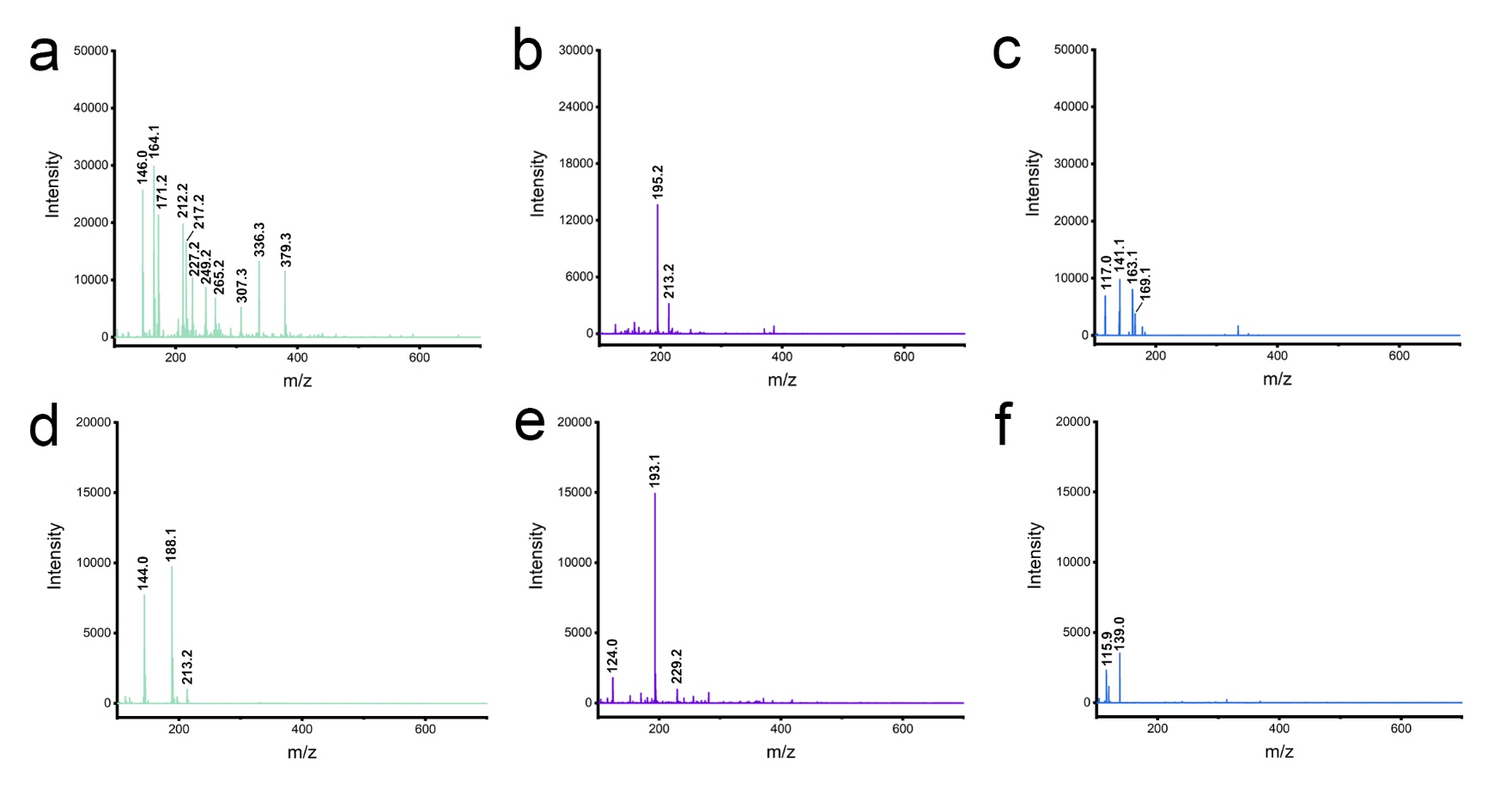


**Figure S3.** MS spectra of (a) CHCA, (b) 9-AA, (c) IVONSs:Sm in positive-ion mode, and (d) CHCA, (e) 9-AA, (f) IVONSs:Sm in negative-ion mode.

To further assess the suitability of nano-matrix IVONSs:Sm in the LDI-MS process, MS spectra of CHCA, 9-AA, and IVONSs:Sm without analyte were collected in dual-polarity modes. It was observed that predominant matrix-related peaks were present for CHCA in positive-ion mode (Figure S3a). In contrast to the production of multiple ions in positive-ion mode, fewer matrix ions of CHCA were detected in the LMW region under negative-ion mode (Figure S3d). Similarly, backgrounds with several intrinsic matrix-related peaks were also observed for those using 9-AA as matrices in different ionization modes (Figure S3b, e). The absence of matrix peaks for IVONSs:Sm ranging from m/z 150 to m/z 600, especially in negative-ion mode, demonstrated the potential of IVONSs:Sm as a stable matrix for LDI-MS analysis (Figure S3c, f).


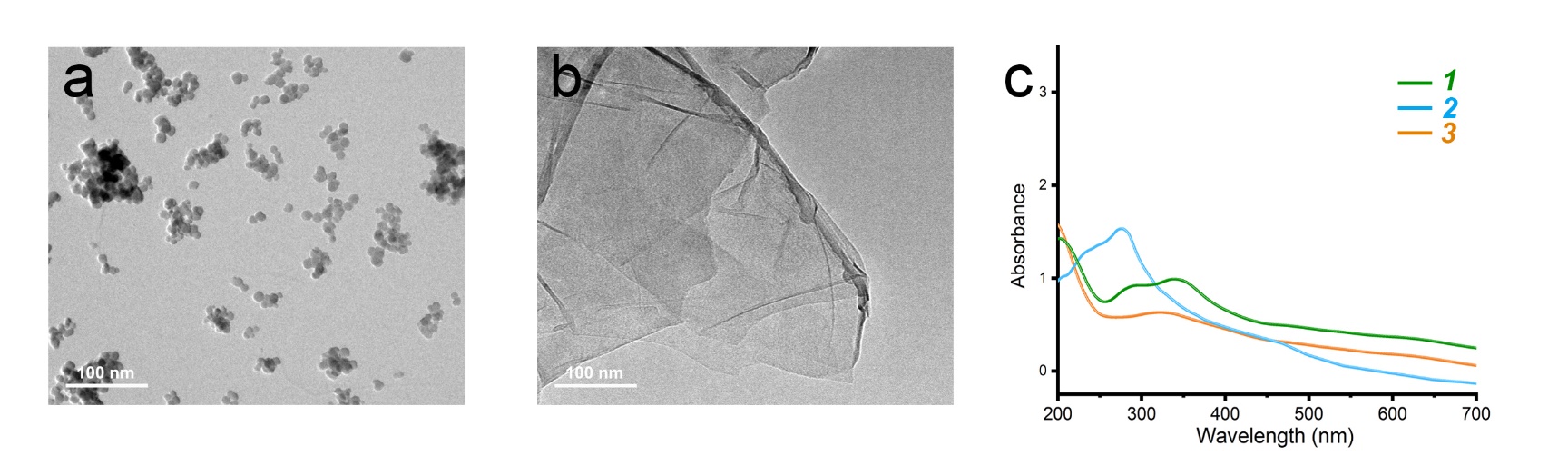


**Figure S4.** TEM images of (a) graphene and (b) CeO_2_ nano-matrices for fatty acids analysis. (c) UV-vis absorption spectra of three nano-matrices: IVONSs:Sm (1), graphene (2), and CeO_2_ (3).

The morphology of graphene and CeO_2_ was characterized by TEM. The TEM characterization of graphene and CeO_2_ was similar to that in previous reports,^[5]^ and the results are also shown in Figure S4a, b. Specifically, Figure S4a showed that the size of the obtained nano-matrix CeO_2_ was distributed in the range from 6 to 15 nm, and the TEM image (Figure S4b) revealed the flake-like structure of graphene. Additionally, the UV-vis absorption spectra of the three nano-matrices with the same dispersing concentration were also investigated. A stronger absorption of IVONSs:Sm than other nano-matrices at the operational wavelength (355 nm) made it possible to be used as a benign receptor of laser energy in the LDI-MS process (Figure S4c).


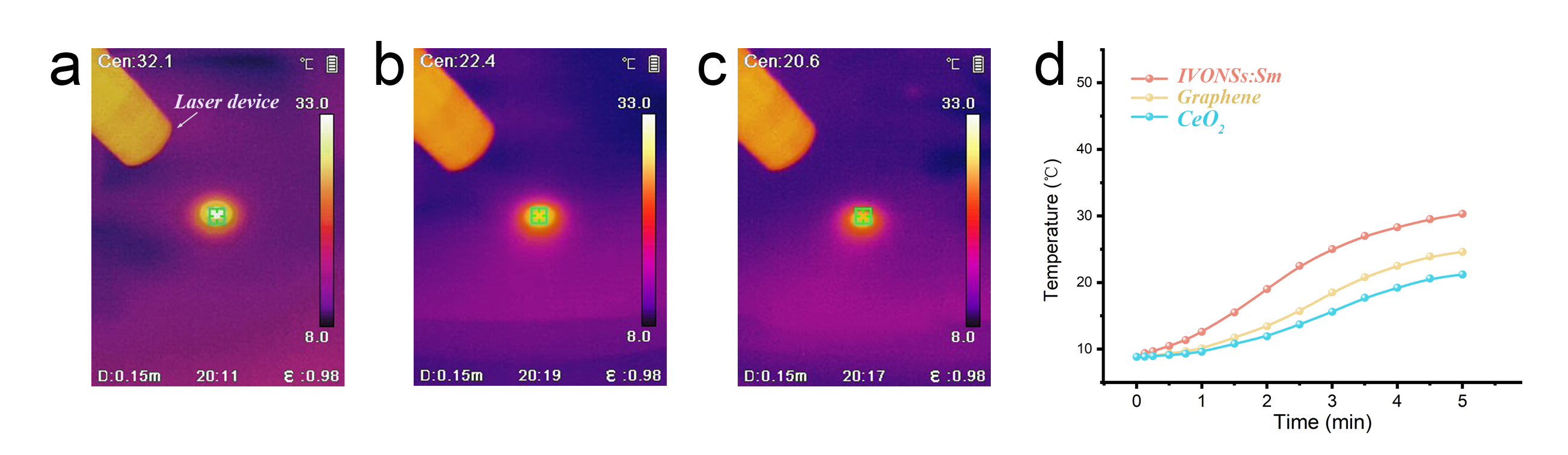


**Figure S5.** Infrared thermal images of (a) IVONSs:Sm, (b) graphene, and (c) CeO_2_ nano-matrices irradiated with 405 nm laser (2 W/cm^2^) for 5 min, respectively. (d) Photothermal conversion curves of the three nano-matrices exposed to 405 nm laser irradiation.

Conventionally, the laser energy absorbed by the matrix is often transferred to heat within the plume, which then ionizes the analytes. Therefore, the photothermal conversion properties of the three nano-matrices were compared by monitoring the temperature variation upon 405 nm laser irradiation at 2 W/cm^2^ for 5 minutes. As shown in Figure S5a~c, the infrared thermographic images of the three nano-matrices were recorded during sequential irradiation. The temperature of the IVONSs:Sm sample reached 32.1 °C, while indistinctive temperature increases under the same conditions were observed for the graphene and CeO_2_ nano-matrices due to the weak absorption and conversion of laser energy. Additionally, photothermal heating curves also confirmed the variation in temperature changes of the aforementioned nano-matrices (Figure S5d). All the results indicated that IVONSs:Sm exhibited more advantageous photothermal conversion properties than other typical nano-matrices, making it effective in enhancing the ionization of analytes.


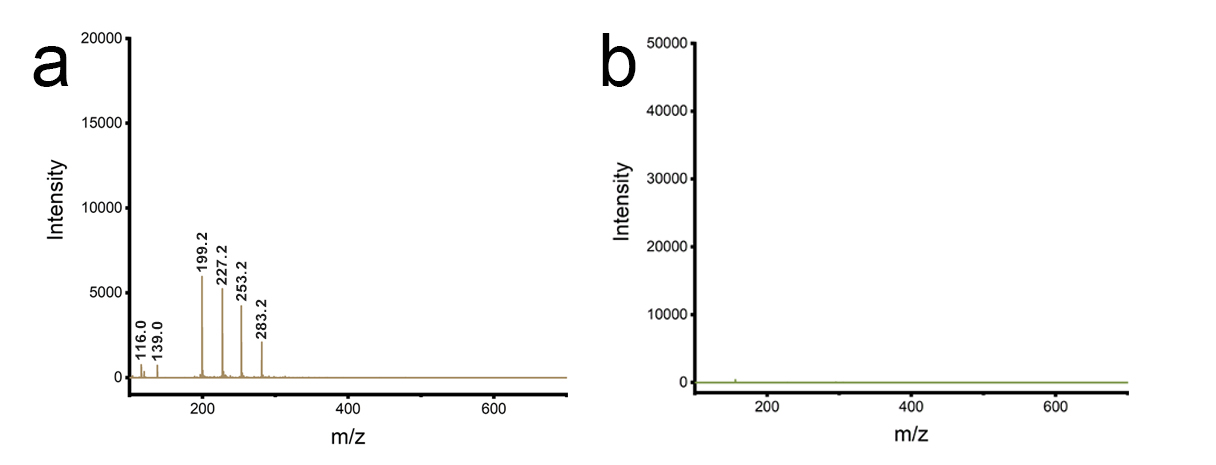


**Figure S6.** (a) MS spectrum of four fatty acids analyzed by using pure IVONSs as nano-matrix. (b) MS spectrum of four fatty acids analyzed by using dopant Sm(NO_3_)_3_ as control.

Figure S6a exhibited a similar negative-ion MS spectrum of four fatty acids using pure IVONSs without samarium doping, but with insufficient MS intensities compared to that using IVONSs:Sm (Figure 2c, e). This indicated that the doping of Sm^3+^ into IVONSs might contribute to the LDI process and efficiency in the negative-ion mode, making IVONSs:Sm more feasible for fatty acid analysis than pure IVONSs. Additionally, the MS spectrum of four fatty acids was tested using dopant Sm(NO_3_)_3_ as the matrix. The absence of MS signal of four fatty acids suggested that the MS signal enhancement of IVONSs:Sm in the negative-ion mode was the result of the synergistic effects of IVONSs and Sm^3+^ (Figure S6b).

**
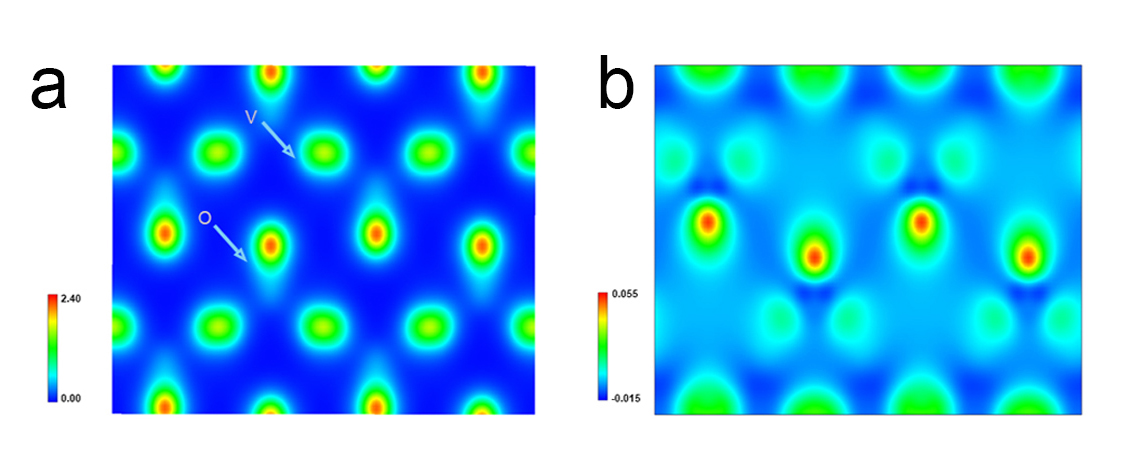
**

**Figure S7.** (a) Electron density profile and (b) differential electron density profile of pure IVONSs.


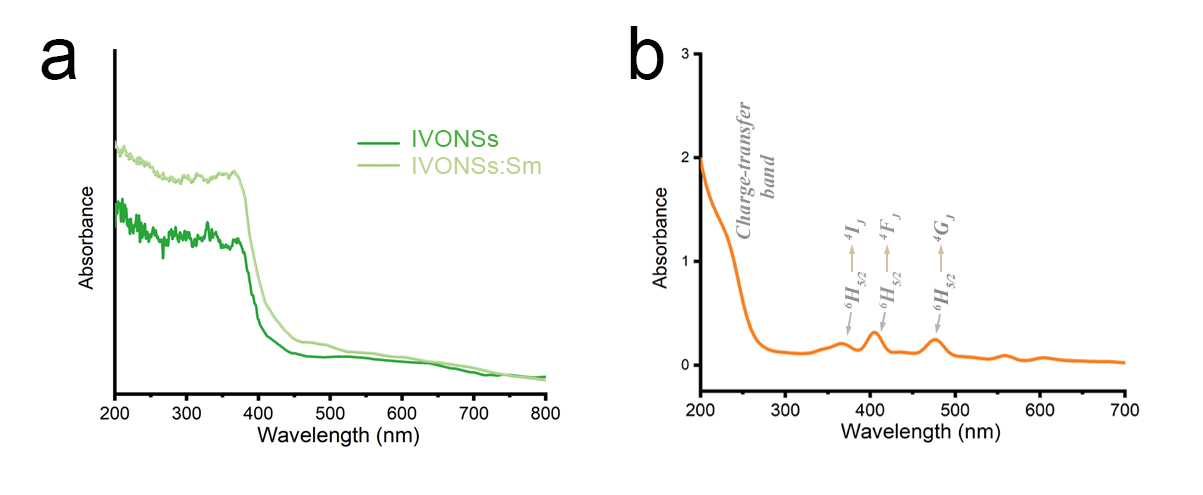


**Figure S8.** Optical absorption behaviors of (a) IVONSs:Sm and pure IVONSs, and (b) dopant Sm(NO_3_)_3_.

Figure S8a depicted the diffuse reflectance ultraviolet-visible spectra of IVONSs:Sm and pure IVONSs. It is worth noting that the absorption bands ranging from 200-400 nm were mainly attributed to the charge transfer effect between V^5+^–O^2-^, covering the most widely used laser wavelength (e.g. 355 nm Nd:YAG laser) in MALDI-MS instruments. Specifically, the absorption band of IVONSs was strengthened and slightly red-shifted after the introduction of Sm^3+^. The absorption spectrum of dopant Sm(NO_3_)_3_ was comparatively probed, exhibiting the featured emissions from f-f transitions of Sm^3+^ (335-500 nm) and the charge-transfer absorption from the nitrate ligands to Sm^3+^ (Figure S8b). This result spectroscopically implied the mechanism of enhanced absorption of IVONSs:Sm, suggesting that IVONSs:Sm were more predominant in the absorption of laser power, allowing for the subsequent ionization process.

**
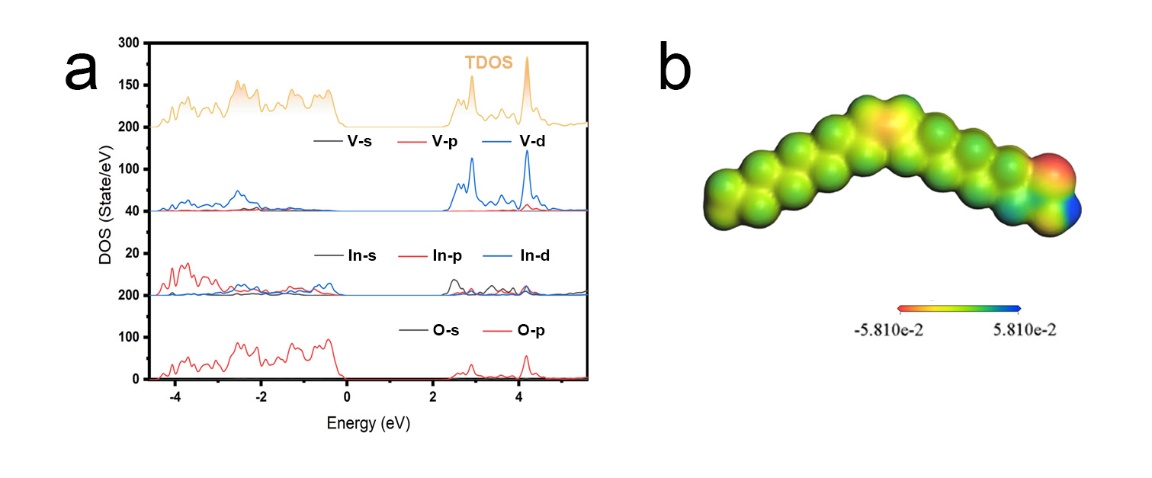
**

**Figure S9.** (a) Calculated DOS of pure IVONSs. (b) Calculated electrostatic potential of OA molecule.

**
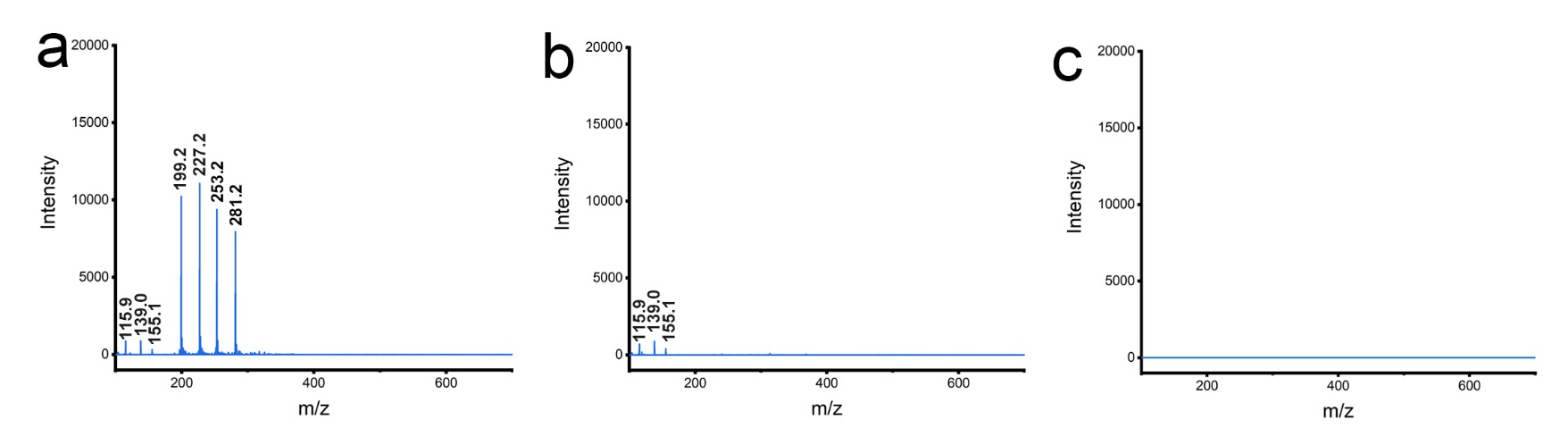
**

**Figure S10.** MS spectra of (a) IVONSs:Sm+fatty acids mixture, (b) IVONSs:Sm, and (c) fatty acids mixture on the copper conductive tape, respectively.

The performance of IVONSs:Sm as the nano-matrix on the copper conductive tape has been investigated, with IVONSs:Sm pipetted onto the copper conductive tape instead of being directly dropped on the MALDI-target plate. Figure S10a showed that a concise signal of analytes could be detected with strong intensities, and there was an interference-free background in the range of m/z 160~700 without the addition of four fatty acids (Figure S10b). It is important to note that there was no signal of analytes in the absence of IVONSs:Sm (Figure S10c), indicating the critical role of IVONSs:Sm in the negative ionization of low molecular weight compounds on the copper conductive tape.


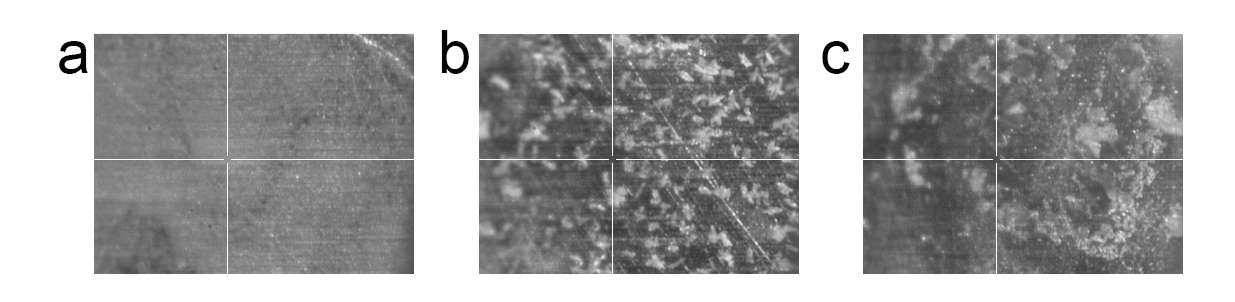


**Figure S11.** Optical images of dried samples spotted with different matrices of (a) IVONSs:Sm, (b) 9-AA, and (c) CHCA.


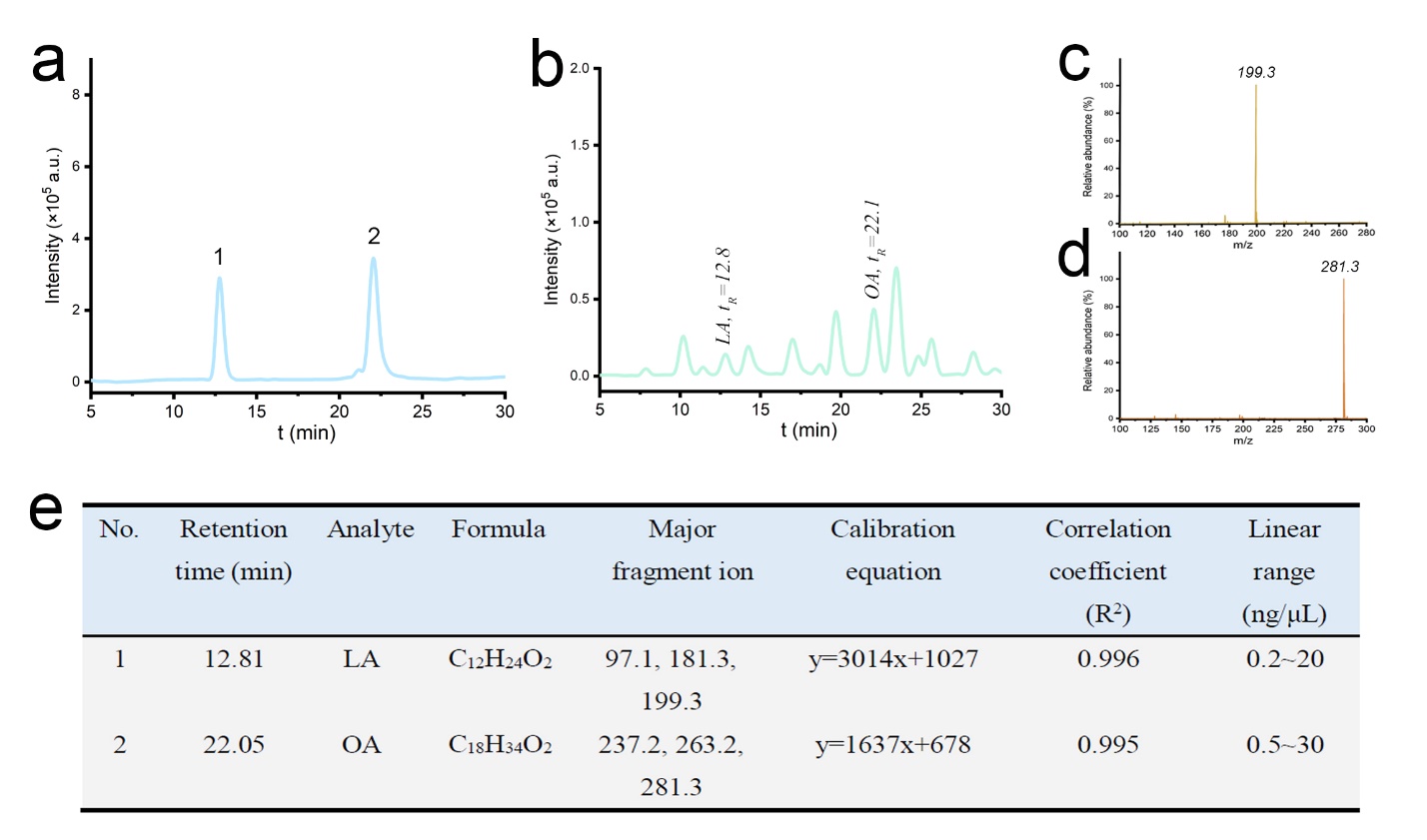


**Figure S12.** (a) Total ion chromatogram of two representative fatty acid standards (LA and OA). (b) Total ion chromatogram of HPLC-ESI-MS for methanol extraction of fingerprint. Mass spectrometric identification of (c) LA and (d) OA in fingerprint extracting solution. (e) Calibration and quantitative results of the two fatty acids.

The quantitative analysis of LA and OA residues in fingerprints was performed using HPLC-ESI-MS. ^[6,7]^ According to the optimized chromatographic conditions (Experimental Section, Supporting Information), the two fatty acid standards appeared as distinct peaks eluted at retention times of 12.81 and 22.05 min, respectively (Figure S12a). Meanwhile, a chromatogram of the methanol extraction of the fingerprint was obtained, and the endogenous LA and OA were identified via retention time and mass spectrometric experiments (Figure S12b). The qualitative identification and quantitative information of the two fatty acids detected in the HPLC-ESI-MS system were summarized in Figure S12c~e. For the methanol extraction of fingerprints (200 μL), the average concentration of LA and OA was determined to be 1.8 ng/μL and 13.6 ng/μL, respectively. Therefore, the residual content of LA and OA in a fingerprint sample was calculated to be 0.36 μg and 2.72 μg, respectively. As depicted in Figure S13a, considering the LODs for LA (8.2 μM) and OA (11.6 μM), including the added volume (1 μL) of fatty acid standard solutions, it was sensitive enough to detect 1.6×10^-3^ μg of LA and 3.3×10^-3^ μg of OA using the proposed IVONSs:Sm-assisted LDI-MS approach in the real samples. Note that these values were much lower than those measured by HPLC-ESI-MS, so the quantitative results further verified that IVONSs:Sm-assisted LDI-MS was adequate to ensure sensitivity for fingerprint analysis.


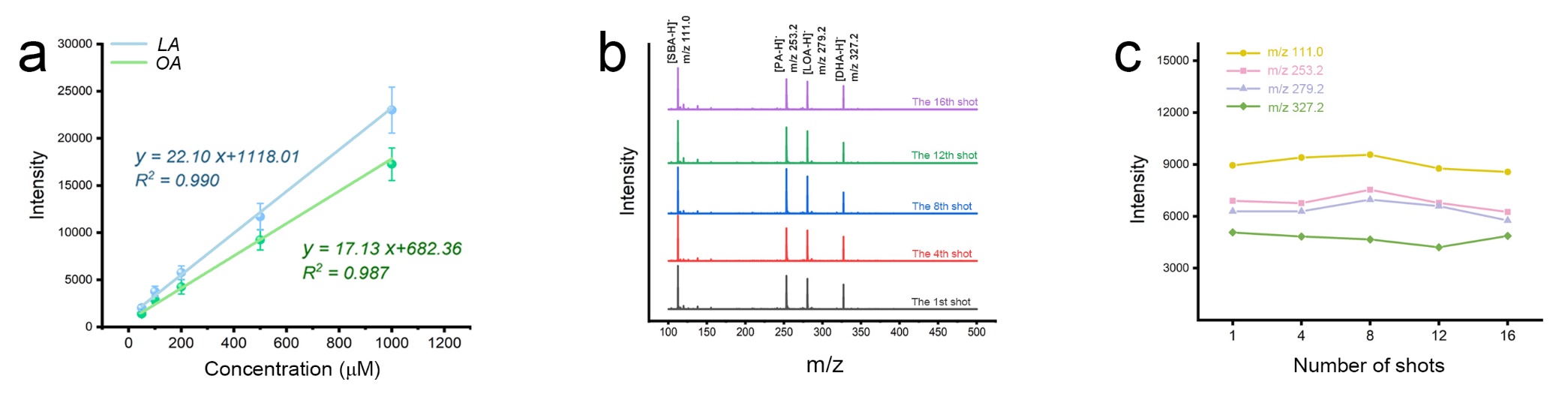


**Figure S13.** (a) Relationships between the analyte concentrations and the MS signal intensities of [M−H]^−^. (b) IVONSs:Sm-assisted LDI-MS spectra and (c) MS intensity changes of four typically active LMW compounds at the same spot after repeated laser shots.

The photostability of analytes during IVONSs:Sm-assisted LDI-MS analysis was assessed. As shown in Figure S13b, IVONSs:Sm-assisted LDI-MS spectra of four typically active LMW compounds, namely sorbic acid (SBA, m/z 111.0), palmitoleic acid (PA, m/z 253.2), linoleic acid (LOA, m/z 279.2), and docosahexaenoic acid (DHA, m/z 327.2), were repeatedly collected from one spot after multiple laser shots. However, the result of the MS spectra implicated that there was barely any change with any emerging new peaks after multiple laser shots, and the MS signal intensities of the four LMW compounds were almost stable (Figure S13c), ruling out possible photocatalytic reactions of organic analytes under the experimental IVONSs:Sm-assisted LDI-MS condition.


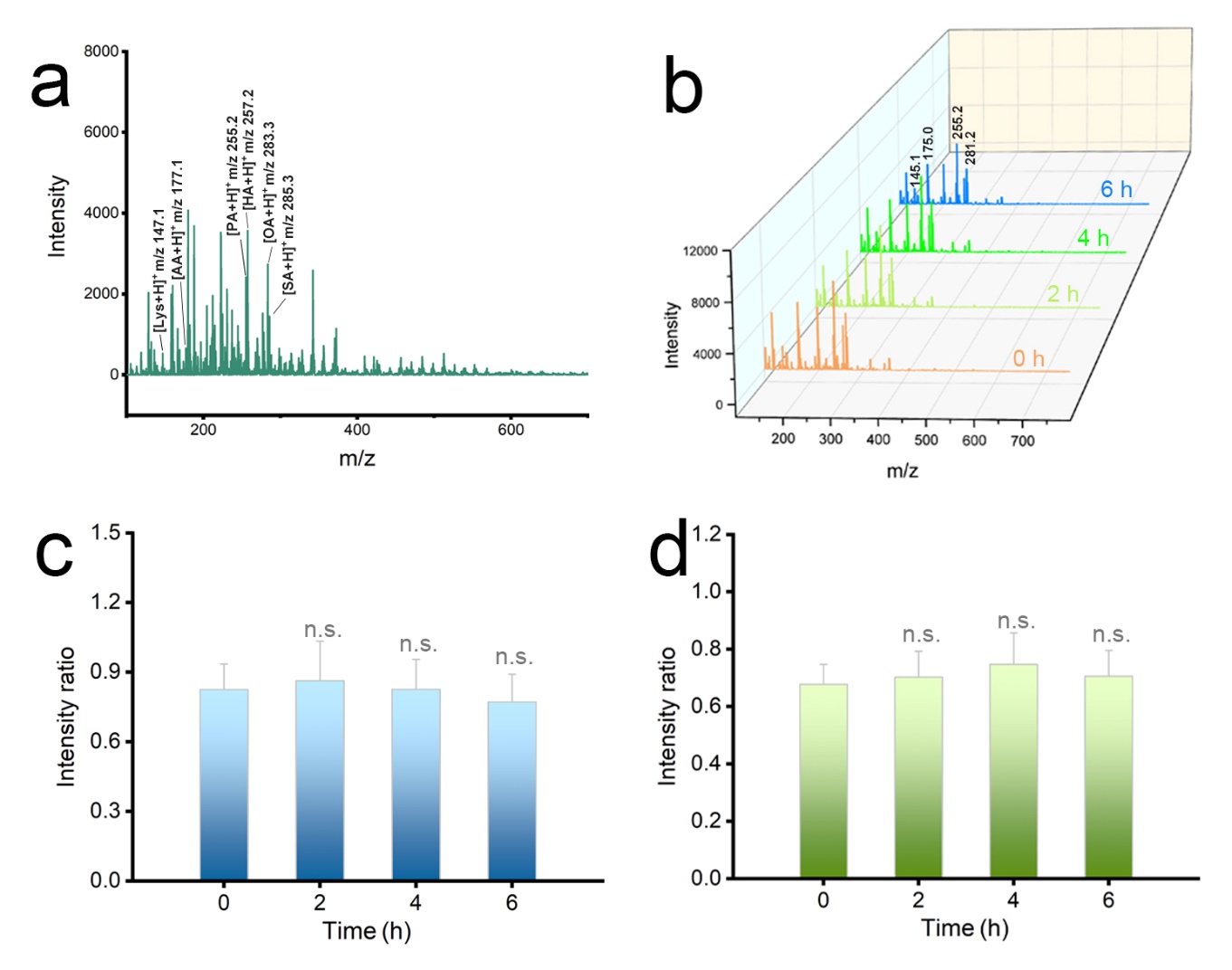


**Figure S14.** (a) Representative IVONSs:Sm-assisted LDI-MS spectrum of fingerprint in positive-ion mode. (b) IVONSs:Sm-assisted LDI-MS spectra of fingerprint sample stored in vacuum with different time in negative-ion mode. MS intensity ratio between (c) OA signal at m/z 281.2 and HA signal at m/z 255.2, (d) AA signal at m/z 175.0 and HA signal at m/z 255.2, respectively.

Given that a longer period of time is needed for IVONSs:Sm-assisted LDI-MS imaging analysis, IVONSs:Sm and the LMW compounds in the fingerprint should remain nonvolatile and stable in high vacuum over the long term. Therefore, the fingerprint samples sprayed with IVONSs:Sm were loaded into the MALDI-MS spectrometer for MS spectra collection at specific time intervals under vacuum conditions. The MS spectra in Figure S14b revealed that the nano-matrix IVONSs:Sm and the LMW compounds in the fingerprint samples could maintain long-term stability with negligible volatility, which was further confirmed by a stable MS intensity ratio between some representative LMW compounds (Figure S14c, d).


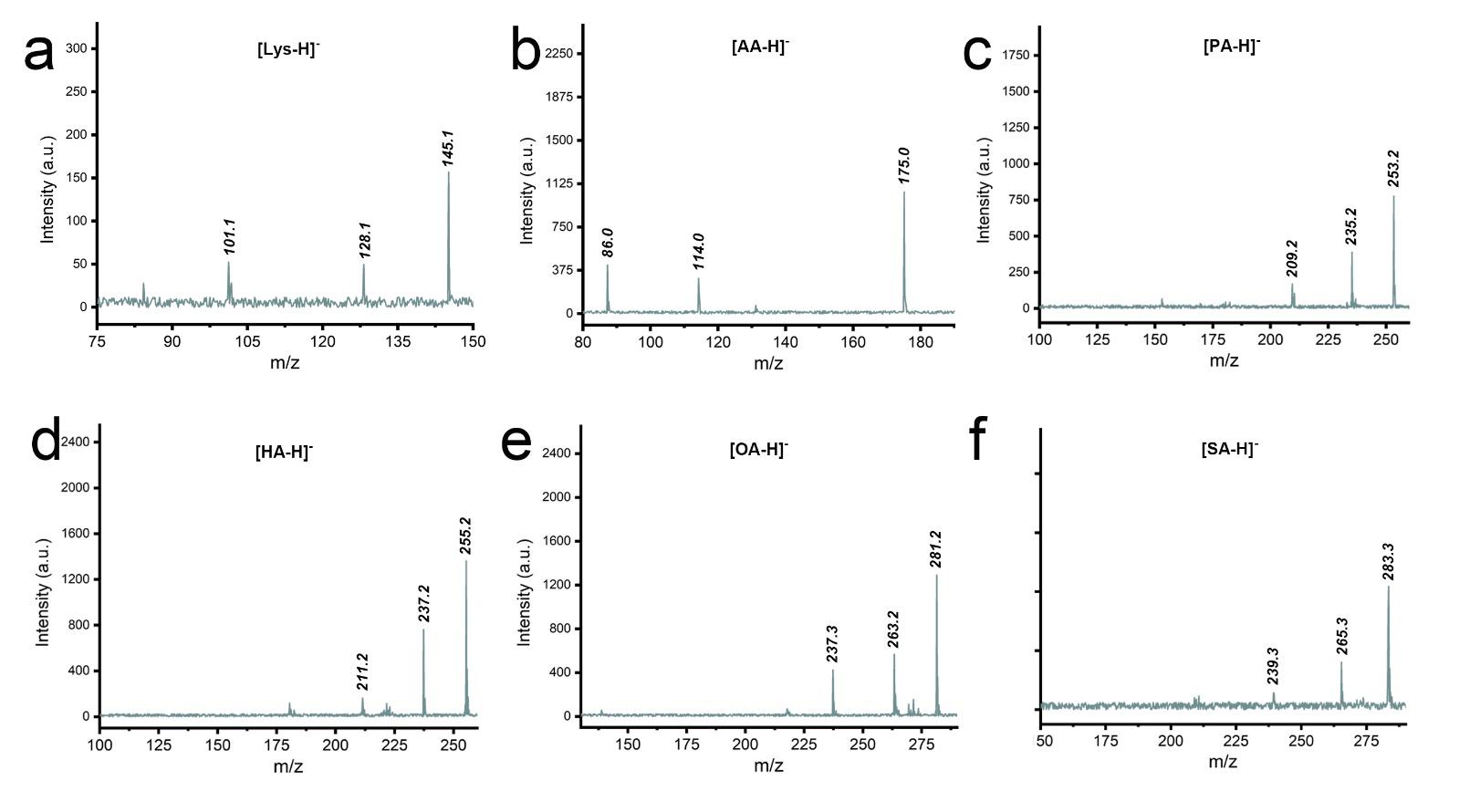


**Figure S15.** The MALDI LIFT-TOF/TOF identification of (a) Lys, (b) AA, (c) PA, (d) HA, (e) OA, and (f) SA from fingerprint samples in negative-ion mode, respectively.

Owing to a low matrix-related background interference and high detection sensitivity, numerous deprotonated LMW compounds were detected from fingerprint samples in negative-ion mode (Figure S15a~f), such as Lys (m/z 145.1, [M−H]^−^), AA (m/z 175.0, [M−H]^−^), PA (m/z 253.2, [M−H]^−^), HA (m/z 255.2, [M−H]^−^), OA (m/z 281.2, [M−H]^−^), and SA (m/z 283.3, [M−H]^−^). Putative identification of intense ions was verified by using MALDI LIFT-TOF/TOF MS, and followed by searching database and comparing with previously reported data. ^[8-12]^ Besides, validation information of other LMW compounds detected in fingerprint samples were listed in Table S3.


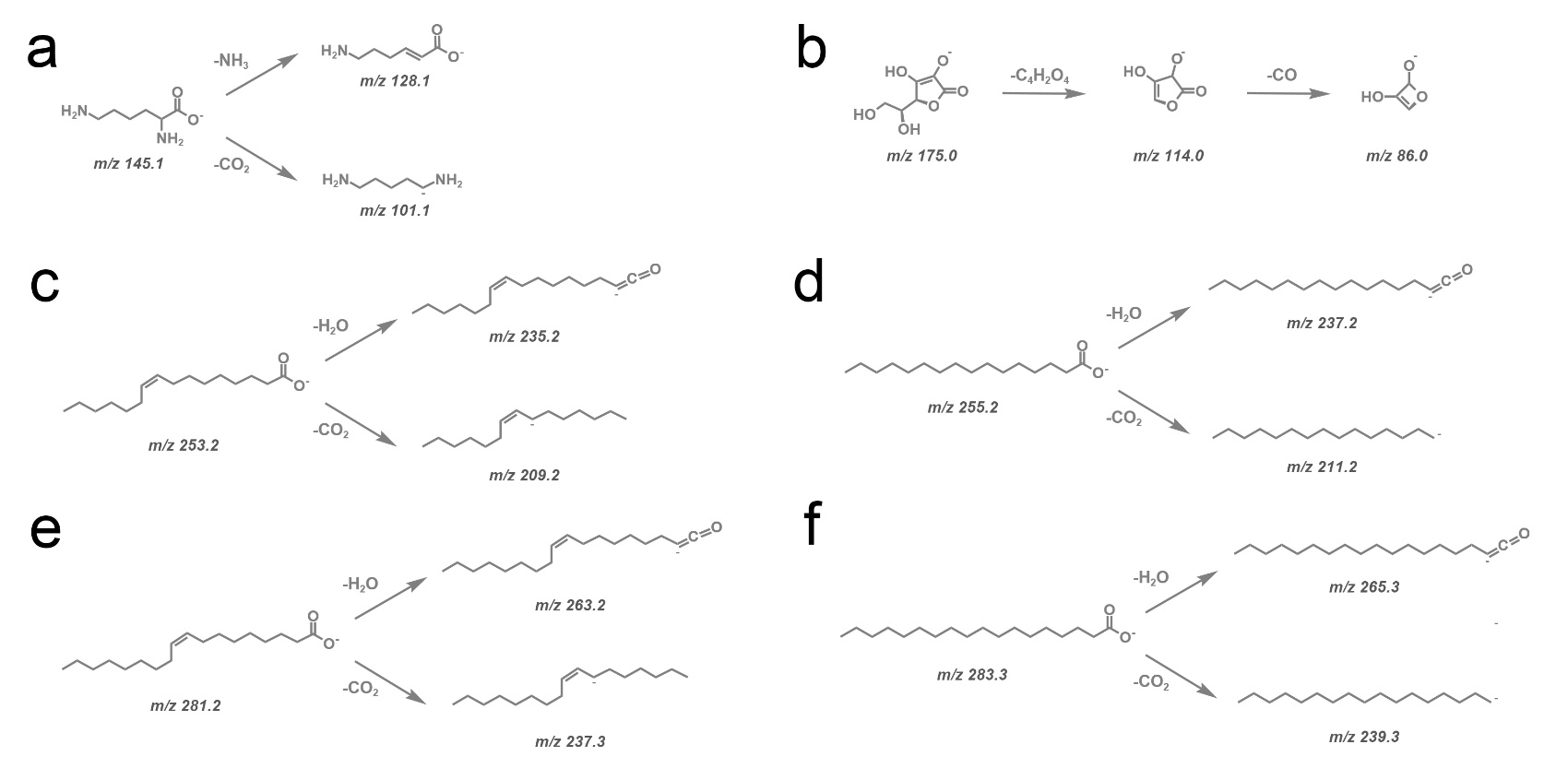


**Figure S16.** The possible mechanism of mass spectral fragmentation for (a) Lys, (b) AA, (c) PA, (d) HA, (e) OA, and (f) SA from fingerprint samples in negative-ion mode, respectively.


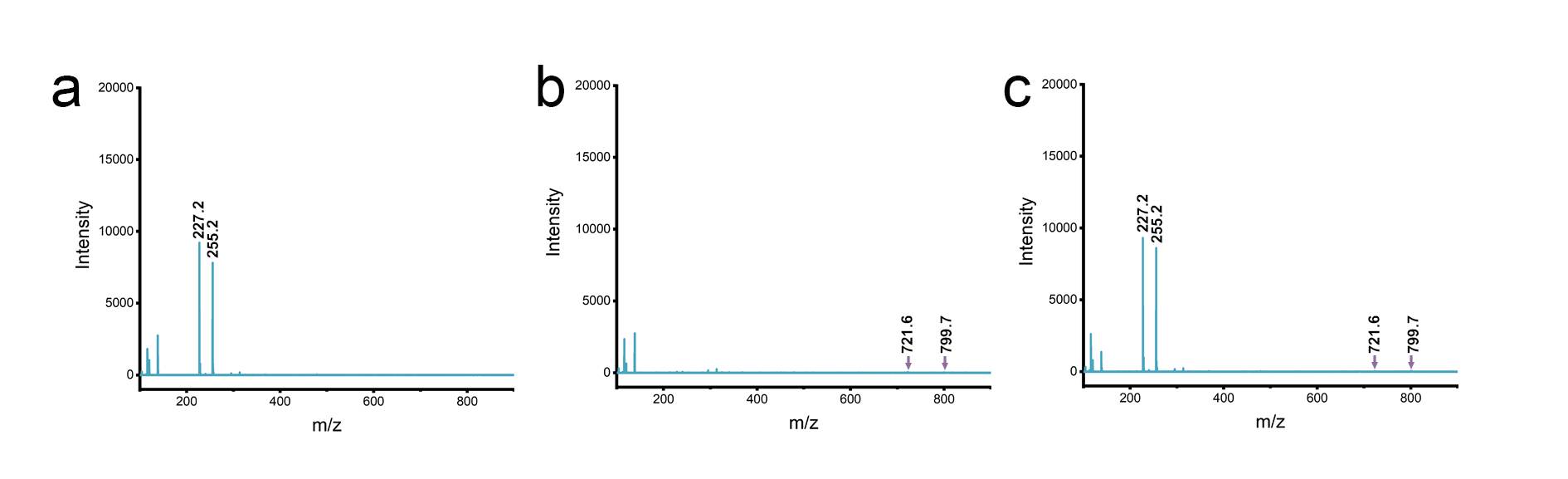


**Figure S17.** IVONSs:Sm-assisted LDI-MS spectra of equal mixture of (a) MA and HA, (b) glycerol trimyristate and glycerol tripalmitate, and (c) two fatty acids and two glycerides in negative-ion mode, respectively.


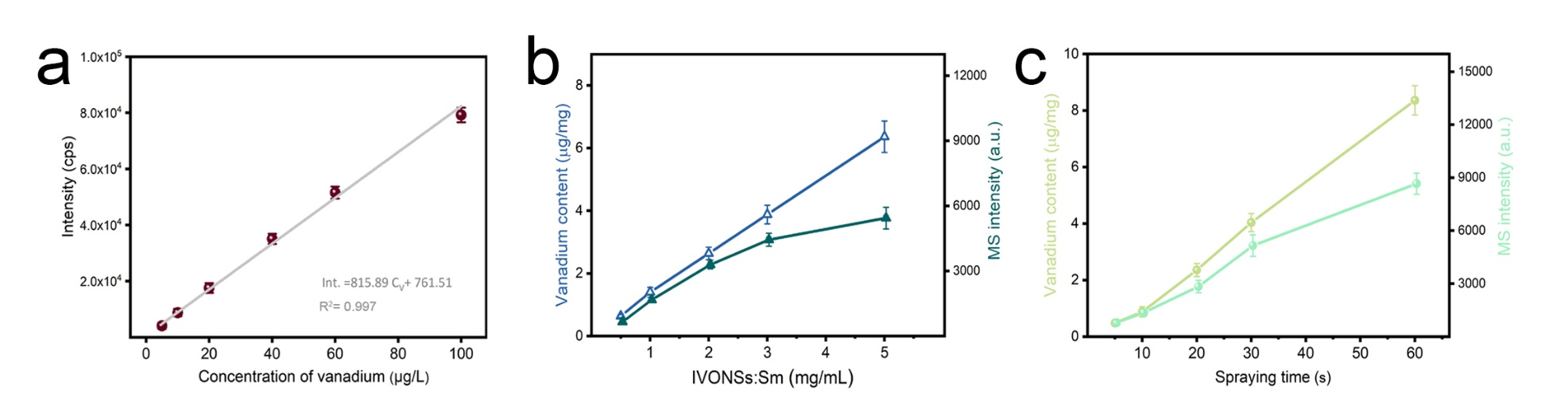


**Figure S18.** (a) Linear relationship between ICP-MS intensity and the concentration of vanadium element. Optimizations of the (b) dispersing concentration and (c) spraying time of the nano-matrice IVONSs:Sm suspensions in fingerprints detection.


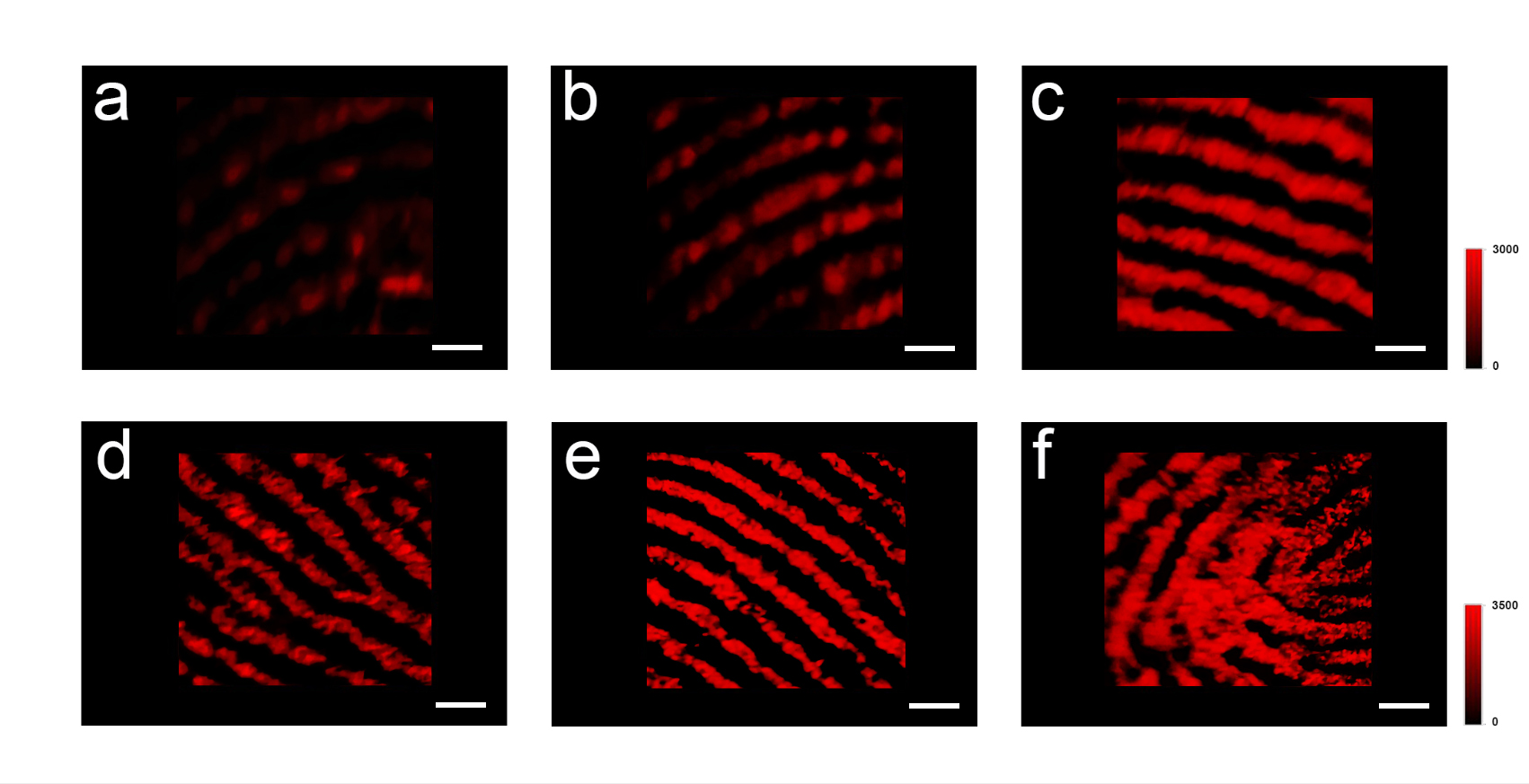


**Figure S19.** Representative IVONSs:Sm-assisted LDI-MS images of fingerprints sprayed with (a) 2 mg/mL, (b) 3 mg/mL, and (c) 5 mg/mL IVONSs:Sm suspension for 30 s, respectively, or sprayed with 5 mg/mL IVONSs:Sm suspension for (d) 20 s, (e) 30 s, and (f) 60 s, respectively. Scale bars represent 1 mm.


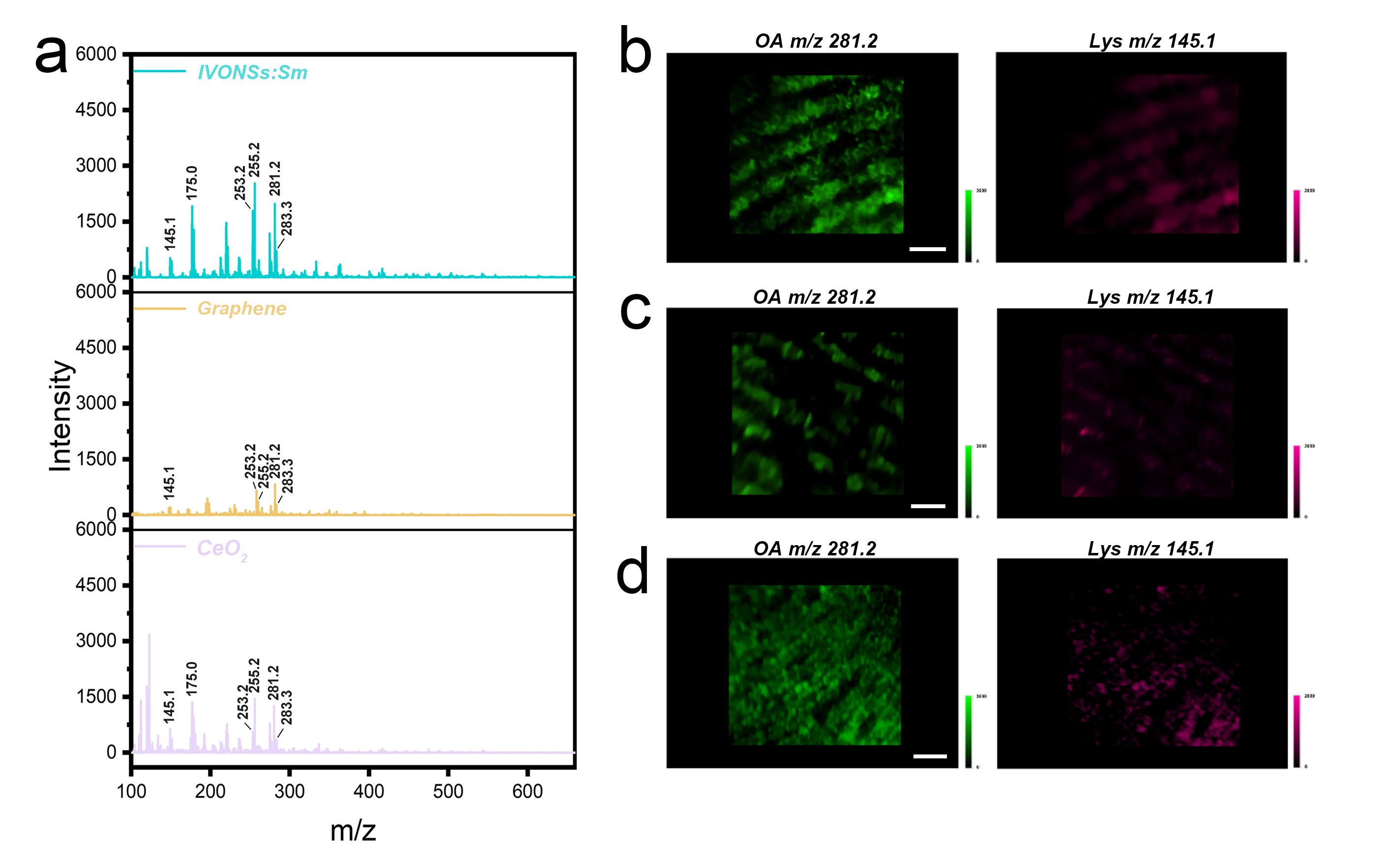


**Figure S20.** (a) IVONSs:Sm-assisted LDI-MS spectra and (b)~(c) MS images of fingerprints using different nano-matrices in negative-ion mode: (b) IVONSs:Sm, (c) graphene, and (d) CeO_2_. Scale bars represent 1 mm.


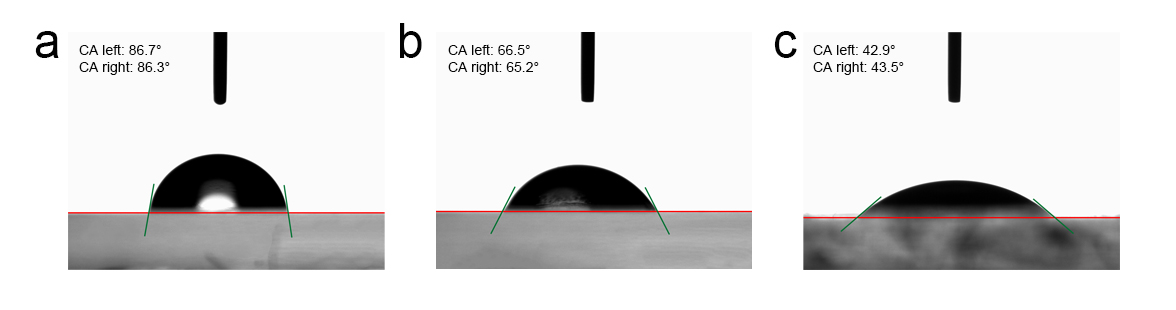


**Figure S21.** Aqueous contact angle measurements of (a) IVONSs:Sm, (b) graphene, and (c) CeO_2_, respectively.

The hydrophobicity analysis for the three nano-matrices was carried out via aqueous contact angle (CA) measurements. As shown in Figure S21a, a more hydrophobic property with a contact angle of 86.5° could be obtained for IVONSs:Sm compared with graphene and CeO_2_ (Figure S21b, c). This property might not only facilitate the homogeneous dispersion of IVONSs:Sm in the suspending medium bromoethane but also enhance the interaction between the nano-matrix and hydrophobic analytes, thus allowing desired fingerprint images with higher contrast between ridges and valleys (Figure S20b). This experiment helped to explain the discrepancy among the imaging effects of LMW compounds using the three nano-matrices (Figure S20b~d).


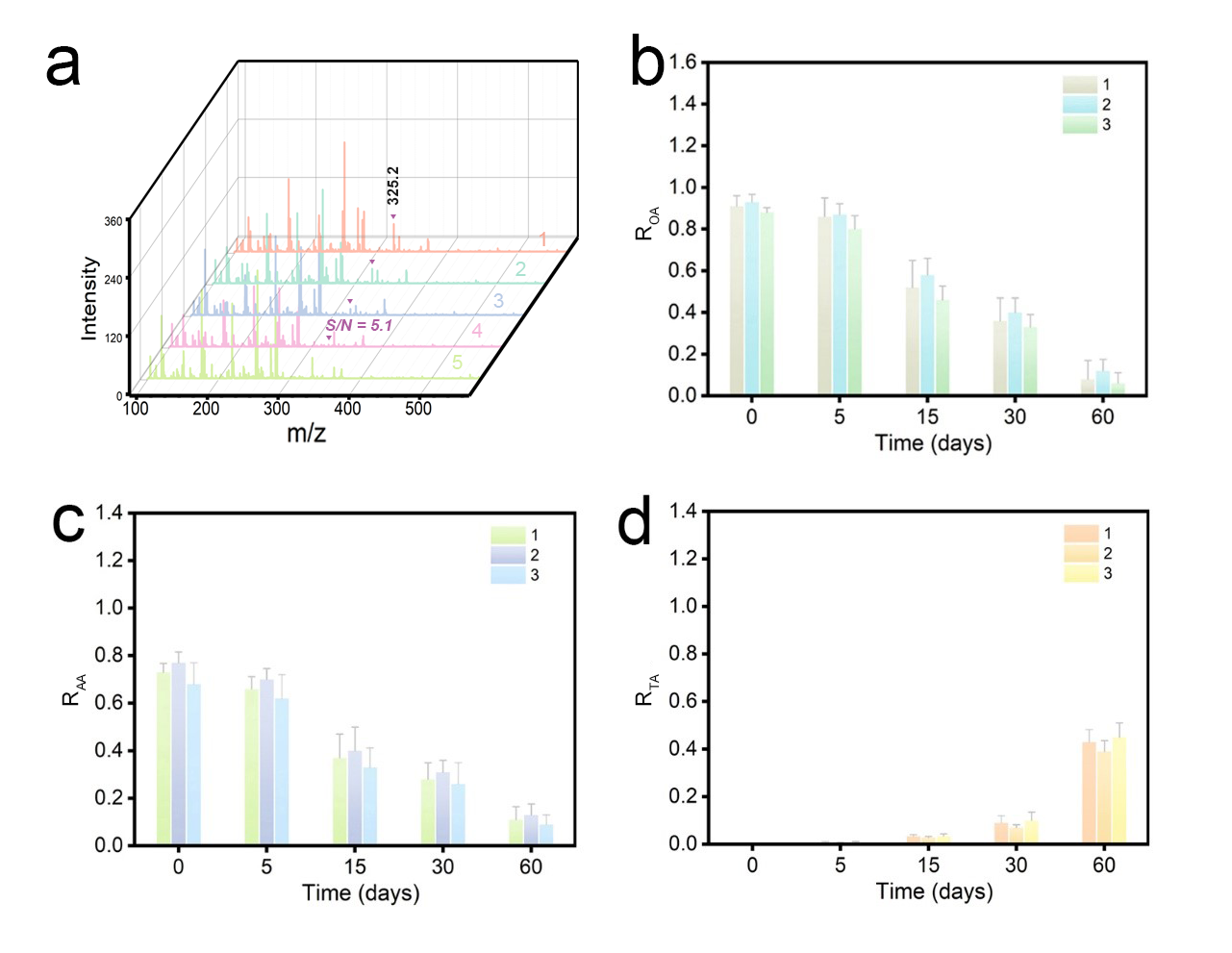


**Figure S22.** (a) IVONSs:Sm-assisted LDI-MS analysis for exogenous SDBS with different content levels on fingerprint samples. The fingerprint samples No.1~5 were collected after washing hands with the diluted lotions of gradient dilution ratio: undiluted (1); 1/2 (2); 1/5 (3); 1/10 (4); and 1/20 (5). Time-dependent MS intensity ratios for (b) OA, (c) AA, and (d) TA measured in three typical environmental conditions: 30 °C/60 % RH/light-free (1); 20 °C/25 % RH/ambient light (2); and 40 °C/90 % RH/ambient light (3).


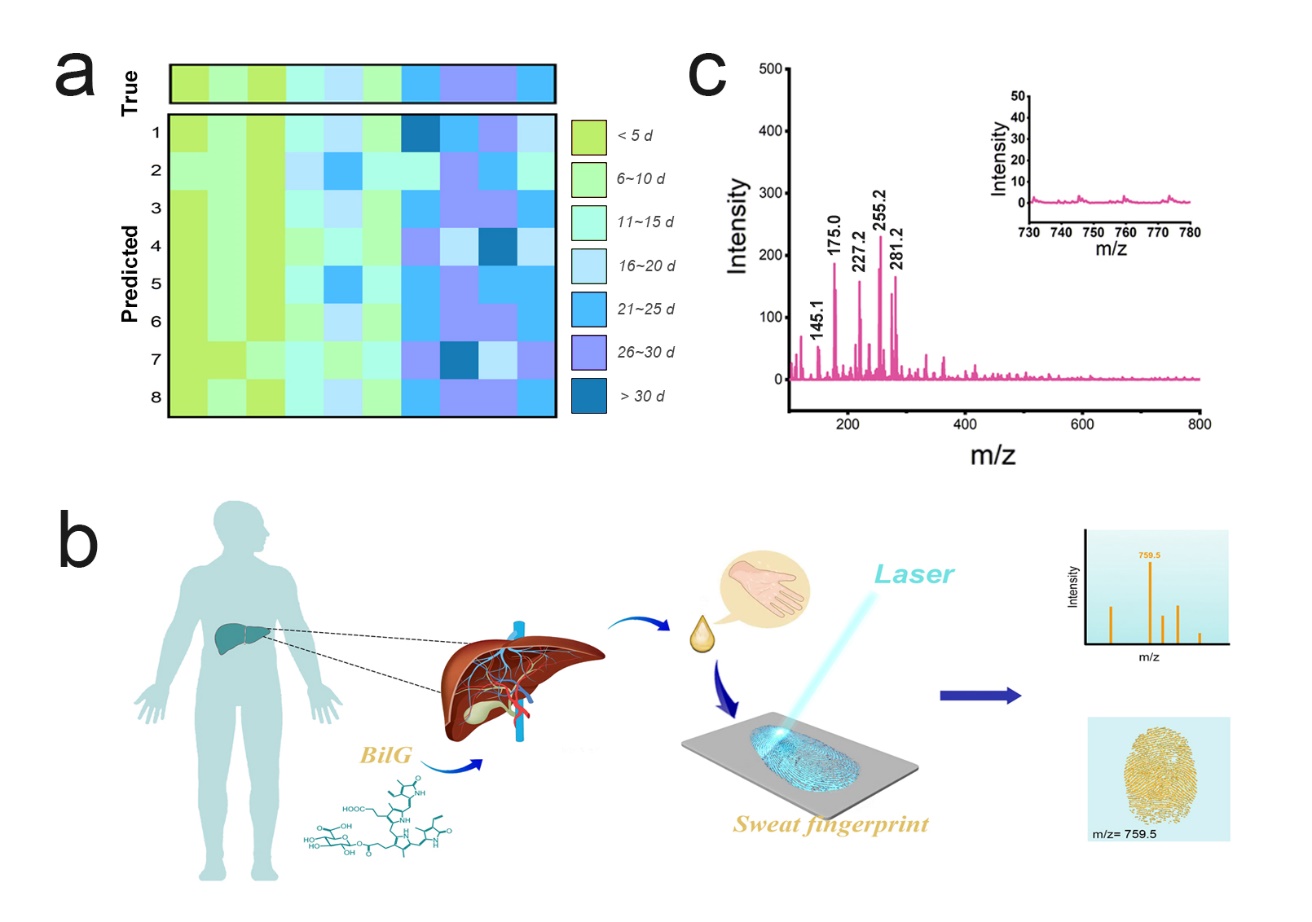


**Figure S23.** (a) Evaluation of the aging time of ten simulated fingerprint specimens based on the time-dependent MS intensity ratios. (b) Illustration of BilG detection in sweat fingerprint using IVONSs:Sm-assisted LDI-MS tool. (c) Representative IVONSs:Sm-assisted LDI-MS spectra of fingerprints from healthy volunteer.


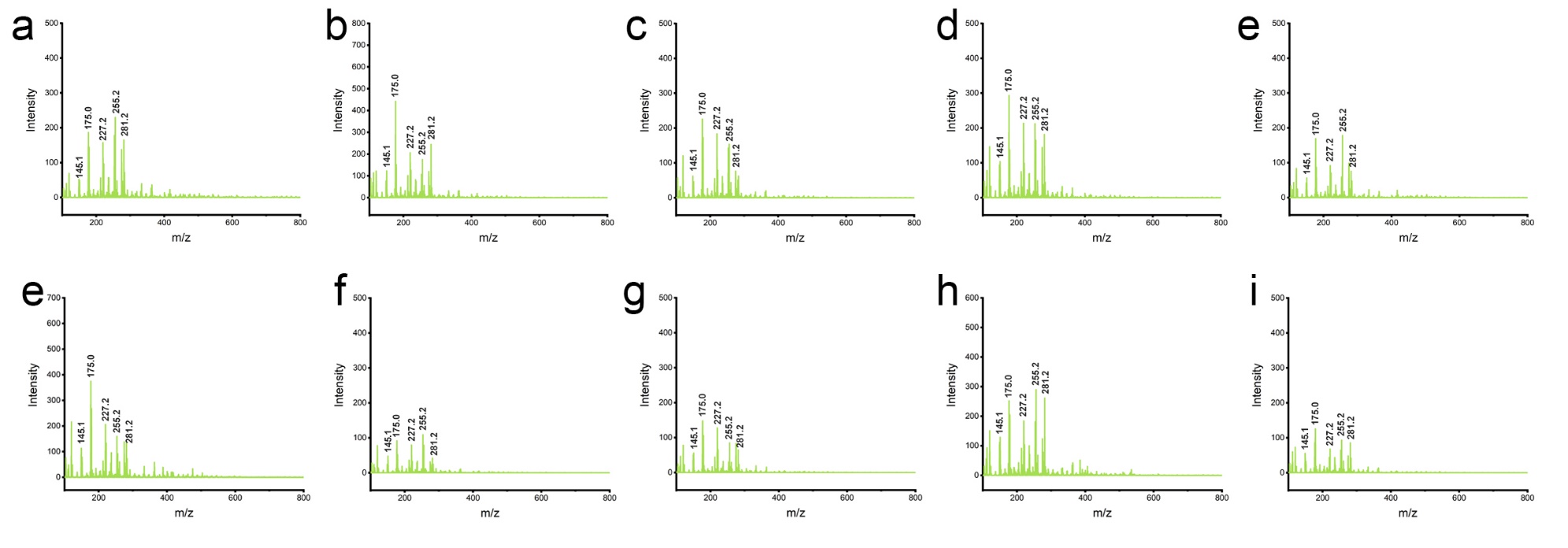


**Figure S24.** (a)~(i) The negative-ion mode MS spectra of fingerprint samples from healthy volunteers No.1~10, respectively.


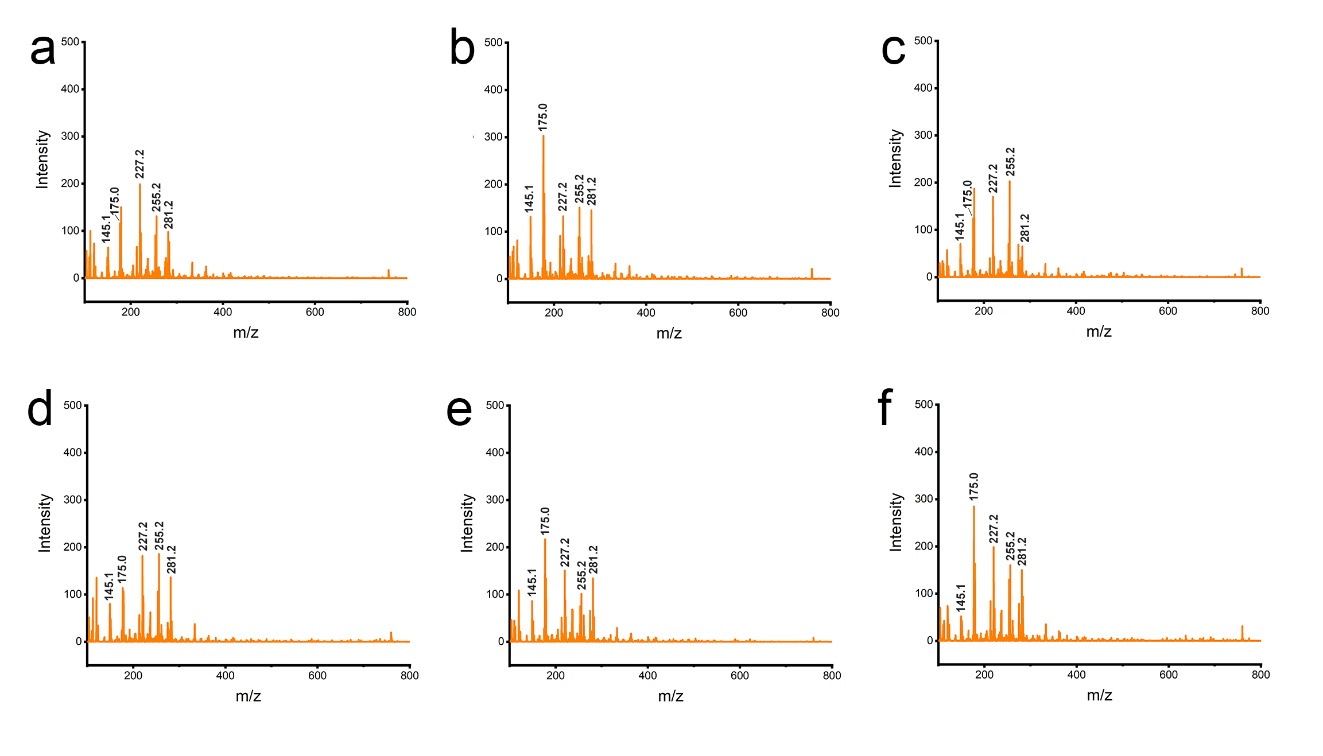


**Figure S25.** (a)~(f) The negative-ion mode MS spectra of fingerprint samples from acute hepatitis patients No.1~6, respectively.

**Table S2.** MS analysis result of four fatty acids using CHCA, 9-AA, or IVONSs:Sm in positive- and negative-ion modes.

| Ion mode | m/z | Detected ions | IVONSs:Sm | CHCA | 9-AA |
| --- | --- | --- | --- | --- | --- |
| **+** | 201.2  223.2  239.3  245.1  229.2  251.2  267.3  273.2  255.2  277.2  293.3  299.2  283.3  305.2  321.3  327.2 | [LA+H]^+^  [LA+Na]^+^  [LA+K]^+^  [LA+2Na-H]^+^  [MA+H]^+^  [MA+Na]^+^  [MA+K]^+^  [MA+2Na-H]^+^  [PA+H]^+^  [PA+Na]^+^  [PA+K]^+^  [PA+2Na-H]^+^  [OA+H]^+^  [OA+Na]^+^  [OA+K]^+^  [OA+2Na-H]^+^ | ★  ★  ★  ★  ★  ★  ★  ★  ★  ★  ★  ★  ★  ★  ★  ★ | ★  ★  ★  ★  ★  ★  ★    ★  ★  ★  ★  ★  ★ | ★  ★  ★  ★  ★  ★    ★  ★  ★  ★  ★  ★ |
|  | Identified ions | | 16 | 13 | 12 |
|  | Missing percentage | | 0 % | 18.7 % | 25.0 % |
|  | Background interfering ions | | 11 | 20 | 12 |
| **-** | 199.2  227.2  253.2  281.2 | [LA-H]^-^  [MA-H]^-^  [PA-H]^-^  [OA-H]^-^ | ★  ★  ★  ★ |  | ★  ★  ★  ★ |
|  | Identified ions | | 4 |  | 4 |
|  | Missing percentage | | 0 % |  | 0 % |
|  | Background interfering ions | | 3 | 10 | 17 |

**Table S3.** Putative identification of components detected by IVONSs:Sm-assisted LDI-MS in negative-ion mode.

| No. | Compound | Formula | Ion form | | Theoretical m/z | Observed m/z | Error △ppm | | MS/MS fragments |
| --- | --- | --- | --- | --- | --- | --- | --- | --- | --- |
| 1 | Palmitoleic acid | C_16_H_30_O_2_ | | [M-H]^-^ | 253.2168 | 253.2208 | | 16 | 209.2, 235.2 |
| 2 | Hexadecylic acid | C_16_H_32_O_2_ | | [M-H]^-^ | 255.2324 | 255.2377 | | 21 | 211.2, 237.2 |
| 3 | Oleic acid | C_18_H_34_O_2_ | | [M-H]^-^ | 281.2480 | 281.2522 | | 15 | 237.3, 263.2 |
| 4 | Stearic acid | C_18_H_36_O_2_ | | [M-H]^-^ | 283.2637 | 283.269 | | 19 | 239.3, 265.3 |
| 5 | Lysine | C_6_H_14_O_2_N_2_ | | [M-H]^-^ | 145.0977 | 145.1032 | | 38 | 101.1, 128.1 |
| 6 | Ascorbic acid | C_6_H_8_O_6_ | | [M-H]^-^ | 175.0243 | 175.0313 | | 40 | 86.0, 114.0 |
| 7 | Oleic acid hydroperoxide | C_18_H_34_O_4_ | | [M-H]^-^ | 314.2457 | 314.2501 | | 14 | 201.1, 215.1, 270.2 |
| 8 | 9-Oxononanoic  acid | C_9_H_16_O_3_ | | [M-H]^-^ | 172.1099 | 172.1168 | | 40 | 100.0, 128.1 |
| 9 | Threonic acid | C_4_H_8_O_5_ | | [M-H]^-^ | 136.0372 | 136.0413 | | 30 | 74.0, 118.0 |
| 10 | Sodium dodecyl benzene sulfonate | C_18_H_29_O_3_SNa | | [M-Na]^-^ | 325.1837 | 325.1872 | | 11 | 183.1, 197.2, 261.2 |
| 11 | Retinoic acid | C_20_H_28_O_2_ | | [M-H]^-^ | 299.2011 | 299.2061 | | 17 | 119.1, 255.2 |
| 12 | Bilirubin glucuronide | C_39_H_44_N_4_O_12_ | | [M-H]^-^ | 759.2878 | 759.2939 | | 8 | 297.1, 566.3 |

**Table S4.** Comparison and properties of different inorganic nano-matrices used for fingerprint LDI-MS analysis.

| No. | Compound | Dimension | λmax | Contact angle | Identified ions number |
| --- | --- | --- | --- | --- | --- |
| 1 | IVONSs:Sm | 0.2~2.5 μm (lateral) | 350 nm | 86° | 59 (negative-ion mode) |
| 2 | Graphene | 0.3~5 μm (lateral) | 270 nm | 66° | 26 (negative-ion mode) |
| 3 | CeO_2_ | 6~15 nm (diameter) | 320 nm | 43° | 38 (negative-ion mode) |

**References**

[1] G. Kresse, J. Furthmüller, *Computational Materials Science* **1996,** 6, 15.

[2] G. Kresse, D. Joubert, *Physical Review B* **1999,** 59, 1758.

[3] S. Grimme, *Journal of Computational Chemistry* **2006,** 27, 1787.

[4] V. Wang, N. Xu, J. C. Liu, G. Tang, W. Geng, *Computer Physics Communications* **2021,** 267, 108033.

[5] Wang, Y. *Analytical Chemistry* **2010,** 82, 6208-6214.

[6] H.Mok, J. Lee, R. Bandu, H. Kang, K. Kim, K. Kim, *RSC Advances* **2016,** 6, 32130.

[7] L. Chen, B. Xie, L. Li, W. Jiang, Y. Zhang, J. Fu, G. Guan, Y. Qiu, *Chromatographia* **2014,** 77, 1241.

[8] C. Ding, L. Wang, Y. Yao, C. Li, *Food Chemistry* **2022,** 392, 133298.

[9] S. Yamabe, N. Tsuchida, S. Yamazaki, S. Sakaki, *Organic & Biomolecular Chemistry* **2015,** 13, 4002.

[10] G. Fuchs, *Annual Review of Microbiology* **2011,** 65, 631.

[11] H. J. Mok, J. W. Lee, R. Bandu, H. S. Kang, K.-H. Kim, K. P. Kim, *RSC Advances* **2016,** 6, 32130.

[12] S. Thurnhofer, W. Vetter, *Journal of Agricultural and Food Chemistry* **2005,** 53, 8896.
